# ADGRL1 is a glucose receptor involved in mediating energy and glucose homeostasis

**DOI:** 10.1007/s00125-023-06010-6

**Published:** 2023-09-15

**Authors:** Kavaljit H. Chhabra, Siresha Bathina, Tumininu S. Faniyan, Dennis J. Samuel, Muhammad Ummear Raza, Leticia Maria de Souza Cordeiro, Gonzalo Viana Di Prisco, Brady K. Atwood, Jorge Robles, Lauren Bainbridge, Autumn Davis

**Affiliations:** 1 Department of Medicine, Division of Endocrinology, Diabetes and Metabolism, University of Rochester School of Medicine and Dentistry, Rochester, NY, USA; 2 Department of Pharmacology and Physiology, University of Rochester Medical Center, Rochester, NY, USA; 3 Department of Pharmacology & Toxicology, Indiana University School of Medicine, Indianapolis, IN, USA; 4 Stark Neurosciences Research Institute, Indiana University School of Medicine, Indianapolis, IN, USA

**Keywords:** Diabetes, Glucose receptor, Glucose sensing, Hypothalamus, Mouse models, Obesity

## Abstract

**Aims/hypothesis:**

The brain is a major consumer of glucose as an energy source and regulates systemic glucose as well as energy balance. Although glucose transporters such as GLUT2 and sodium–glucose cotransporter 2 (SGLT2) are known to regulate glucose homeostasis and metabolism, the identity of a receptor that binds glucose to activate glucose signalling pathways in the brain is unknown. In this study, we aimed to discover a glucose receptor in the mouse hypothalamus.

**Methods:**

Here we used a high molecular mass glucose–biotin polymer to enrich glucose-bound mouse hypothalamic neurons through cell-based affinity chromatography. We then subjected the enriched neurons to proteomic analyses and identified adhesion G-protein coupled receptor 1 (ADGRL1) as a top candidate for a glucose receptor. We validated glucose–ADGRL1 interactions using CHO cells stably expressing human ADGRL1 and ligand–receptor binding assays. We generated and determined the phenotype of global *Adgrl1-*knockout mice and hypothalamus-specific *Adgrl1*-deficient mice. We measured the variables related to glucose and energy homeostasis in these mice. We also generated an *Adgrl1*^Cre^ mouse model to investigate the role of ADGRL1 in sensing glucose using electrophysiology.

**Results:**

*Adgrl1* is highly expressed in the ventromedial nucleus of the hypothalamus (VMH) in mice. Lack of *Adgrl1* in the VMH in mice caused fasting hyperinsulinaemia, enhanced glucose-stimulated insulin secretion and insulin resistance. In addition, the *Adgrl1*-deficient mice had impaired feeding responses to glucose and fasting coupled with abnormal glucose sensing and decreased physical activity before development of obesity and hyperglycaemia. In female mice, ovariectomy was necessary to reveal the contribution of ADGRL1 to energy and glucose homeostasis.

**Conclusions/interpretation:**

Altogether, our findings demonstrate that ADGRL1 binds glucose and is involved in energy as well as glucose homeostasis in a sex-dependent manner. Targeting ADGRL1 may introduce a new class of drugs for the treatment of type 2 diabetes and obesity.

## Introduction

Glucose is a major source of energy for the brain [1]. The brain in turn regulates systemic glucose and energy homeostasis by responding to changes in blood glucose levels. For example, hypoglycaemia increases hunger in animals to signal them to increase their food intake and consequently restore glucose as well as energy balance [2, 3]. Conversely, hyperglycaemia under normal conditions decreases hunger [4, 5]. Most of the research regarding how our brain regulates this homeostasis focuses on glucose metabolism or its utilisation, probably because the biology of glucose transport and its consequent biochemical pathways is well established. The concept of glucoreceptors involved in glucose sensing and glucose signalling pathways independently of glucose metabolism has been proposed for quite some time [6–9]. Although glucose transporters such as GLUT2 and sodium–glucose cotransporter 2 (SGLT2) regulate glucose homeostasis, including glucose sensing [10, 11], their involvement in direct glucose signalling pathways dissociated from their role in glucose transport or metabolism is unclear [12, 13]. In addition, not all glucose-sensing neurons express ATP-sensitive potassium channel (K_ATP_) channels [14] or glucokinase [15, 16], which are traditionally associated with glucose-sensing mechanisms. Furthermore, K_ATP_ deficiency does not completely abolish glucose sensing in the hypothalamus [14, 17–19]. Taste type 1 receptors are also suggested to sense glucose and contribute to glucose homeostasis [20], but whether these receptors bind glucose and trigger glucose signalling pathways is unknown. Overall, glucose metabolism and classical glucose-sensing mechanisms alone do not completely explain glucose signalling in the hypothalamus.

Little progress has been made in our basic understanding of whether or how glucose signals via its plasma membrane receptors to influence whole-body glucose and energy balance. In the 1950s, J. Mayer proposed the glucostatic theory and possibility of existence of a central glucoreceptor [7, 8, 21] to explain how blood glucose levels might regulate hunger. Yet, the identity of a receptor that binds with glucose and is involved in glucose signalling pathways is unknown. Therefore, here we addressed this longstanding research question of identifying plasma membrane glucose receptors that are likely involved in glucose-sensing and glucose-signalling pathways.

Commercially available labelled glucose conjugates (whether they are biotin labelled or radiolabelled) are low molecular mass compounds and they do not provide enough density or sensitivity to isolate glucoreceptors. To overcome this challenge, we synthesised a relatively high molecular mass (30 KDa) glucose conjugate, glucose–biotin–polyacrylamide (PAA), containing 80% glucose, 5% biotin and 15% PAA molecules. We used this conjugate to isolate a glucose receptor. We have characterised interactions between glucose and its receptor using a stable cell line, surface plasmon resonance imaging (SPRi) and recombinant receptors. Finally, we established the function of the receptor in regulating energy and glucose homeostasis using mouse models.

## Methods

Sample randomisation was performed using a GraphPad program (https://www.graphpad.com/quickcalcs/randomize1.cfm). Experimenters were blinded to the groups included in image analyses and microscopy. Blinding was not feasible in the mouse studies because some experimental groups of mice were obese and, therefore, such groups could be identified based on appearance. None of the samples were excluded from the data analyses except in cases of incorrect injection sites of viral vectors after stereotaxic surgery in mice or death of mice.

All animal procedures were approved by the Institutional Animal Care and Use Committee at the University of Rochester or University of Massachusetts and were performed according to the US Public Health Service guidelines for the humane care and use of experimental animals. Mice were housed in ventilated cages under controlled temperature (~23°C) and photoperiod (12 h light/dark cycle, lights on from 06:00 to 18:00 hours) conditions with free access to Hydropac water (Lab Products, USA) and regular laboratory chow (5010, LabDiet, USA).

### Synthesis of glucose–biotin–PAA to isolate glucose receptor

Commercially available labelled glucose conjugates have relatively low molecular mass (200–500 Da) and/or an inadequate proportion of glucose (<20 mol%) to provide sufficient density for ligand–receptor interaction by affinity chromatography. To overcome this problem, we synthesised a 30 KDa conjugate with 80 mol% glucose (ESM Fig. 1) using a published protocol [22] (GlycoNZ, New Zealand). We then used 2.5 mmol/l glucose conjugate to isolate a glucoreceptor. Binding of the glucose or control conjugates to their targets was detected by well-established biotin–streptavidin chemistry using assays described below.

### Magnetic activated cell sorting followed by proteomics

We dissected the hypothalamus from ten C57BL/6J male mice (000664, The Jackson Laboratory; see ‘Mouse models’ below for details of ethics, housing and husbandry relating to all mice used in this study) and dispersed them into single cells using a papain dissociation system (LK003150, Worthington Biochemical Corporation, USA) according to the manufacturer’s instructions. Non-neuronal cells were depleted from the hypothalamic cell suspension using a neuron enrichment procedure according to the manufacturer’s instructions (130-115-389, Miltenyi Biotec, USA). We then divided the neuronal cell suspension equally into two microcentrifuge tubes and added either glucose–biotin–PAA (equivalent to 2.5 mmol/l glucose) or its control (without glucose) to the tubes. The hypothalamic cells were incubated with the conjugates for 30 min at room temperature with constant rotation. After this incubation, the cells were washed with TBS Tween (TBST) for 5 min with gentle rotation and then centrifuged at 200 *g* for 5 min. The supernatant was discarded, and the cells were incubated with anti-biotin microbeads according to the manufacturer’s instructions to enrich biotin-labelled (glucose conjugate or its control) cells by magnetic activated cell sorting (MACS; 130-090-485, Miltenyi Biotec). We further performed three rounds of enrichment for both the glucose- and control polymer-bound neurons. The enriched neurons were lysed with G-protein coupled receptor (GPCR) extraction buffer (A43436, Thermo Fisher Scientific, USA) and subjected to proteomics analyses.

For mass spectrometry analyses of the enriched proteins, trypsin-digested peptides from both the groups were injected onto a homemade 30 cm C18 column with 1.8 μm beads (Sepax, USA), with an Easy nLC-1200 HPLC (Thermo Fisher Scientific), connected to a Fusion Lumos Tribrid mass spectrometer (Thermo Fisher Scientific). Raw data was searched using the SEQUEST search engine within the Proteome Discoverer software platform, version 2.4 (Thermo Fisher Scientific), employing the SwissProt *mus musculus* database. The Minora node was used to determine relative protein abundance between the samples using the Summed Abundance default settings. Percolator 3.5 (https://github.com/percolator/percolator/releases/tag/rel-3-05) was used as the false discovery rate (FDR) calculator, filtering out peptides that had a *q* value greater than 0.01.

### Production of stable cell line, ligand–receptor binding and signalling assays

We transduced 60–70% confluent Chinese hamster ovary (CHO)-K1 cells (CCL-61, ATCC, USA) cultured in a 6 well plate with a lentiviral vector containing human adhesion G-protein coupled receptor L1 (ADGRL1; GLV2-CMV-h*ADGRL1*-puro (3.97×10^8^ infectious units per ml) or its control empty vector (SC1394, GenScript, USA) at multiplicity of infection of 10 in the presence of polybrene (TR-1003, Sigma, USA). We then cultured the cells in F-12K medium (30-2004, ATCC) containing 10% FBS (10099-141C, Gibco, USA) and 10 μg/ml puromycin (A11138-02, Gibco) to generate a polyclonal pool of stable cells expressing human *ADGRL1* or its control. Cells that did not express *ADGRL1* died in the presence of puromycin because they did not have the puromycin resistance cassette. After culturing the pool of the stable cells in a 96 well microplate, we selected the single cell clone with the highest expression of *ADGRL1*. *ADGRL1* overexpression in the cells was measured using qRT-PCR with these primers: forward primer: 5′-AACCAGGTGGCCCAGAAGATC-3′; reverse primer: 5′-CAGCAGCTGCTCCATCAGCTT-3′. The CHO cells were authenticated and tested for absence of mycoplasma contamination at GenScript, USA.

To verify glucose–ADGRL1 binding, we seeded 25,000 CHO-K1 cells expressing either human ADGRL1 or its control per well into 96 well microplates. The microplates were then placed in a sterile incubator that maintained 37°C temperature and 95% relative humidity/5% CO_2_. One day after seeding the cells, we removed the microplates from the incubator, placed them in an aseptic biological cabinet, and washed the cells with Hanks’ Balanced Salt Solution (HBSS; without glucose) twice before equilibrating the cells in the HBSS at 37°C for 1 h. After this equilibration step, the HBSS was removed, and the cells were incubated with different concentrations of the biotinylated glucose or its control (prepared in HBSS without glucose) for 30 min at 4°C. Because ADGRL1 is a membrane receptor, we did not permeabilise the cells to minimise the transport of the conjugates into the cells. The cells were then quickly washed with HBSS twice before adding 100 μl of 10% neutral buffered formalin solution to fix and cross-link the cells for 10 min at room temperature. After this step, the cells were rinsed twice with TBST and streptavidin–horseradish peroxidase (HRP; N100, 1:10,000 dilution, Thermo Fisher Scientific) was added into the wells to react with the cells for 30 min at room temperature. Next, the cells were rinsed twice with TBST and o-phenylenediamine dihydrochloride substrate solution (34006, Thermo Fisher Scientific) was added into the wells to react with the streptavidin–HRP. This substrate yields a yellowish colour with an absorbance maximum at 492 nm measured by a microplate reader. The absorbance values reflect the interaction between ADGRL1 and the biotinylated glucose or its control.

For characterising dynamic interactions between glucose and ADGRL1, we measured the association rate constant (K_on_), dissociation rate constant (K_off_), and the equilibrium dissociation (affinity) constant (K_D_) using SPRi (Horiba XelPlex, Germany). We immobilised the biotinylated glucose conjugate or its control (100 μmol/l) on an extravidin CSe biochip as per the manufacturer’s instructions. The CHO-K1-*ADGRL1* cells were then injected undiluted (5 ×10^5^ cells/ml) and diluted at 1/10, 1/5 and 1/2.5 in HBSS without glucose. For each injection, the number of cells per ml was divided by the Avogadro number 6.0×10^23^ to obtain the molar concentration. Consequently, injected concentrations included in the analysis were 8.3×10^−16^ mol/l, 3.32×10^−16^ mol/l and 1.66×10^−16^ mol/l. Each experiment was performed in triplicate using different cell samples. To further validate glucose–ADGRL1 interaction, we had also immobilised the cells on the biochips coated with 100 μmol/l glucose or its equimolar control conjugate and then injected 18, 36, 71 and 142 nmol/l conjugates in soluble form. Throughout the SPRi, we used the following reagents: cell suspension buffer, HBSS without glucose; running buffer, 10 mmol/l PBS, pH 7.4; blocking buffer, 10 μg/ml biotin + 1% BSA; regeneration buffer, 100 mmol/l NaOH; flow rate, 50 μl/min; sample volume injected through the fluidic system, 200 μl. We generated dose response curves for both the experimental and control CHO-K1 cells to optimise the conditions for measuring K_D_. We had immobilised the glucose or its control conjugate using two different ways—SPRi-Arrayer (contact spotter) and SPRi-CFM (continuous flow microspotter for printing biomolecules)—according to the manufacturer’s instructions. We found that the contact-spotting approach was more optimal than the microfluidic printing with the reagents we had used in this experiment (ESM Fig. 2c). Therefore, we used the spotter for immobilising the biotinylated conjugates and consequently measuring K_D_. The SPRi signals obtained with the control conjugate were used for referencing and normalisation of the results. The kinetic curves were analysed using EzFit 1.4.3 software (Horiba Scientific) to quantify K_on,_ K_off_ and K_D_.

To determine which signal transducers and second messengers are involved in glucose–ADGRL1 signalling, we measured cAMP or Ca^2+^ levels in the stable CHO cell line following glucose treatments. We cultured 10,000 cells/well in 96 well microplates and evaluated the coupling of glucose–ADGRL1 signalling with the Gαs or Gαi proteins using Cisbio Gαs (62AM4PEB, USA) and Gαi (62AM9PEB) assay kits. cAMP in these assays was measured using the homogeneous time-resolved fluorescence method according to the manufacturer’s instructions (PHERAstar FSX plate reader, Bmg Labtech, USA). To assess the involvement of Gαq protein, we used a fluo-4 NW calcium assay kit (F36206, Invitrogen, USA) and measured Ca^2+^ levels using a FLIPR Tetra High-Throughput Cellular Screening System (Molecular Devices, USA) according to the manufacturer’s instructions.

### Production of recombinant human ADGRL1 and glucose–ADGRL1 binding assay

We used a wheat germ cell-free expression system to produce His-tagged human ADGRL1 protein (MPCFK-3, Uniprot ID: O94910, Creative Labs, USA). ADGRL1 proteoliposomes were obtained from this system by centrifugation at 25,000 *g* at 4°C, for 10 min. After removal of the supernatant, the proteoliposome pellets were washed three times with PBS. The pellets were resuspended in 200 μl PBS after the final washing step. Then the ADGRL1 proteoliposomes were analysed for their binding with glucose or stored at −80°C.

For covalent immobilisation of regular (non-conjugated) glucose, we used epoxy activated 96 well plates (695251, PolyAn, Germany). We coated the wells with different concentrations of glucose made in 0.1 mol/l carbonate buffer and 0.15 mol/l NaCl (pH 12 adjusted with NaOH) by incubating the glucose solution in the wells at room temperature for 16 h on a shaker. Control wells received only the vehicle (PBS) without glucose. After the overnight incubation, the wells were washed twice with TBST and 2 μg recombinant ADGRL1 was added to each well for 30 min at room temperature to assess its binding with glucose. Next, the wells were washed twice with TBST. For detection of glucose–ADGRL1 binding using gel electrophoresis, we added 20 μl Laemmli SDS sample buffer to the wells and rinsed them with this buffer for 5 min to solubilise/denature ADGRL1. These samples were then loaded onto a gel to detect ADGRL1 using Coomassie stain. For colorimetric detection of glucose–ADGRL1 binding, we added HRP-conjugated anti-His antibody (ab1187, 1:5000 dilution in TBST, Abcam, USA) and incubated the plate at room temperature for 30 min. After the incubation, the wells were rinsed twice with TBST and o-phenylenediamine dihydrochloride substrate solution (34006, Thermo Fisher Scientific) was added into the wells to react with the HRP-conjugated anti-His antibody and produce a yellowish colour with an absorbance maximum at 492 nm. The absorbance values reflect binding between ADGRL1 and glucose or its control.

### RNA in situ hybridisation, quantitative reverse transcrip‑ tion PCR (qRT‑PCR) and microscopy

We studied the distribution of *Adgrl1* mRNA in the mouse hypothalamus using RNA fluorescence in situ hybridisation. We used the mouse *Adgrl1* probe (319331, ACD, USA) and followed the manufacturer’s instructions to detect its mRNA. We also used mouse *Sf1* (also known as *Nr5a1*; 445731, ACD) and *NeuN* (also known as *Rbfox3*; 313311, ACD) probes to determine their co-localisation with *Adgrl1* in the ventromedial nucleus of the hypothalamus (VMH). DAPI was used to stain the nucleus and verify the regions of interest in the mouse brain. Images were captured using Keyence fluorescence microscope BZ-X800. USA. To further quantify the number of cells expressing *Adgrl1*, *Sf1*, *NeuN* or DAPI, we used CellProfiler 4.4.1 (https://cellprofiler.org/releases).

We used the following primers to measure gene expression using qRT-PCR: *Adgrl1*: 5′-CCATCAAGCAGA ACAGCCGCAA-3′ and 5′-GCTTCACTGTGGCATTCT CCGT-3′, *Hprt*: 5′-AACAAAGTCTGGCCTGTATCC-3′ and 5′-CCCCAAAATGGTTAAGGTTGC-3′. All primers were used at a final concentration of 500 nmol/l. The relative quantity of each mRNA was calculated from standard curves and normalised to the internal control *Hprt*, and then normalised to the mean of corresponding controls. To collect the VMH for qRT-PCR studies, mouse brain was flash frozen and sliced using a cryostat up to −1.4 mm, anterior–posterior, from bregma and the VMH (the third ventricle was used as a landmark) was punched out bilaterally using a blunt 18G needle.

### Mouse models

We produced global *Adgrl1* knockout (KO) mice (Taconic/Cyagen, USA) in which *Adgrl1* transcription is blocked by inserting loxP-En2 SA-PGK-Neo-6*SV40 pA-loxP cassette into intron 4 of the *Adgrl1* gene using CRISPR/Cas-mediated genome engineering. Cas9 and gRNA were co-injected into fertilised eggs with targeting vector for production of these mice. F0 founder mice were identified by PCR and sequence analysis. The mice were then bred with wild-type (WT) mice to test germline transmission and generate F1 mice. These mice were bred with WT C57Bl/6J mice for at least six generations to obtain heterozygotes followed by production of homozygous mice on the C57Bl/6J background. The following primer pairs were used for genotyping the mice: 5′-AACACTTGTATGGCCTTGGGCG-3′ and 5′-AGGCCACTTGTGTAGCGCCA-3′, 5′-TTGAGGCTAGGTGGCATCGCAG-3′ and 5′-CTGCAGTCATGGTTGCTTGGTC-3′. A band at 297 bp indicated the loxP flanked allele and at 268 bp indicated the WT allele. These global *Adgrl1* KO mice have the potential to express normal *Adgrl1* upon Cre exposure in a tissue-dependent manner.

In addition to the global *Adgrl1* KO mice, we generated an *Adgrl1*^loxP/loxP^ mouse model (Ingenious Targeting Laboratory, USA) and induced Cre-mediated *Adgrl1* deficiency in a tissue-specific manner at a desired time to establish the contribution of *Adgrl1* in glucose and energy homeostasis. The *Adgrl1* gene (ENSMUST00000141158.7) was conditionally engineered to be a knockout upon an inversion of the reported trap SA-Exon3*-T2A-tdTomato-BGHpA cassette and Cre expression. The trap cassette was cloned in the open reading frame of exon 3 and it captured the splicing from exon 2. It was flanked by Lox71 and Lox66 elements ensuring a single and irreversible inversion. A long homology arm of ~5.3 kb and short homology arm of ~1.8 kb guided the vector locus integration. Ten microgram of the targeting vector was linearised and then transfected by electroporation of FLP C57BL/6 (BF1) embryonic stem cells (ES). After selection with G418 antibiotic, surviving clones were expanded for PCR analysis to identify recombinant ES clones. The Neo cassette in the targeting vector was removed during ES clone expansion. Targeted iTL BF1 (C57BL/6 FLP) ES cells were microinjected into Balb/c blastocysts (Ingenious Targeting Laboratory) and resulting chimeras with a high percentage black coat colour were mated to C57BL/6N WT mice to generate germline Neo deleted mice. Founder mice (F0) were bred with WT C57Bl/6J mice for at least six generations to obtain heterozygotes followed by production of homozygous mice (*Adgrl1*^loxP/loxP^) on the C57Bl/6J background. The following primers were used for genotyping the mice: 5′-TCAGGTGGATTGAGGTGTTTACCG-3′ and 5′-GGCCTGCAGAACAGTTGTAGACAGTG-3′. A band at 413 bp indicated the loxP flanked allele and 373 bp indicated the WT allele.

After genotyping, mice were randomly assigned to different experimental groups. Randomisation was performed using a GraphPad program (https://www.graphpad.com/quickcalcs/randomize1.cfm)

### Stereotaxic surgery in mice

We used stereotaxic equipment (1900, Kopf, USA) to inject AAV-Cre or -GFP (500 nl, 8×10^12^ vg/ml, University of North Carolina Vector Core) into the VMH (coordinates from bregma: −1.4 mm, anterior–posterior; ±0.4 mm medial–lateral; −5.7 mm dorsal–ventral) of *Adgrl1*^loxP/loxP^ mice and consequently knock down *Adgrl1* in this region or produce a corresponding littermate control group. We studied the phenotype of *Adgrl1*^VMH^-deficient mice at least 3 weeks after the administration of the viral vectors. Unless otherwise noted, ADGLR1 deficiency was induced in 8-week-old mice, after which their phenotype was studied at different times as described in the figure legends. Mice that showed ADGRL1 deficiency in regions other than the VMH, because of inaccurate AAV-Cre injections, were excluded from the study.

### Ovariectomy

To remove both the ovaries in female mice, we anaesthetised them with isoflurane (1–5%), shaved their hair off the flank area, and made a midline incision (~1 cm, mouse placed in prone position) in the region between the last rib and hips. We then separated the musculature using blunt forceps and carefully pulled the ovarian fat pad out of the incision using blunt tweezers followed by clamping the region below the ovary. After isolating the ovarian fat pad, we used a sterile thread and made two knots to identify the area to be removed. We then made a cut just above the knots to remove the ovaries. We verified haemostasis before allowing the uterus to return to the abdomen. The skin incision was closed with sterile sutures. Mice recovered from this surgery within 3–5 days and their weekly body weight was recorded throughout the study.

### Food intake, body composition, physical activity, and glucose and insulin tolerance tests

For food intake measurements, mice were housed individually and fed pre-weighed ad libitum chow. Food consumption was then measured by weighing the remaining food on the 7th day after initiating this experiment. To determine glucose-mediated effects on 24 h food intake, we administered glucose solution (500 mg glucose dissolved in 300 μl water per mouse) at 16:00 and 17:00 hours by oral gavage (18-gauge needle, FNC-18-2-2, Kent Scientific, USA) into *Adgrl1*^VMH^-deficient mice and their littermate controls. For the fasting–refeeding experiment, mice were fasted overnight for 16 h (17:00 to 09:00 hours), after which they were allowed to consume food ad libitum. We also determined the effects of intra-VMH glucose administration (5 mmol/l, 2 μl in PBS over 3 min, administered at 16:00 and 17:00 hours) on feeding. We used a brain infusion kit (3280PD/V/SPC, Plastics1, USA) to implant a bilateral guide cannula into the VMH using the stereotaxic coordinates described above. The cannula was connected to a Hamilton syringe using PE-50 tubing during glucose administration.

Whole-body fat and lean mass were measured using 1H-MRS (Echo Medical System, USA) according to the manufacturer’s instructions. Awake mice were placed in metabolic cages (TSE Systems, USA) to simultaneously measure their energy expenditure, physical activity and oxygen consumption/carbon dioxide production using the manufacturer’s instructions. The energy expenditure data was analysed using the NIDDK Mouse Metabolic Phenotyping Centers (MMPC) ANCOVA program available on http://www.mmpc.org/shared/regression.aspx (accessed 17 May 2022). The mice were acclimatised to the metabolic cages for 3 days before measuring the above-mentioned variables for three additional days.

For glucose tolerance tests, we administered equal amount of glucose (60 mg glucose dissolved in 300 μl water, oral gavage by 18-gauge needle, FNC-18-2-2, Kent Scientific) in mice regardless of differences in their body weight. This was done to prevent obese mice receiving a higher amount of glucose relative to the control mice, and the former being misdiagnosed as having impaired glucose tolerance, as explained in previous publications [23, 24].

We determined insulin sensitivity at different times during the study using insulin tolerance tests, during which we injected insulin (0.75 U/kg body weight, i.p.) and measured blood glucose at baseline, 15, 30, 60 and 120 min after insulin administration.

### Vagotomy

To accomplish selective pancreatic vagotomy, we performed a laparotomy and severed the coeliac vagus nerve of the vagal branches innervating the pancreas. The abdominal cavity was exposed, with the intestine, spleen and stomach gently moved to the right side, to reveal the coeliac nerve branching from the dorsal subdiaphragmatic vagal trunk running along the coeliac artery. The coeliac vagal branches were transected using fine forceps. In control mice, we performed sham surgery using an identical procedure, but the nerves were left intact.

### Hyperinsulinaemic–euglycaemic clamps and glucose uptake study

We performed hyperinsulinaemic–euglycaemic clamps [25, 26] (NIDDK MMPC, UMass Chan Medical School, USA) in awake mice to validate our findings obtained from the insulin tolerance tests. Following the equilibration period, a 2 h hyperinsulinaemic–euglycaemic clamp was conducted with a primed (150 mU/kg body weight) and continuous infusion of insulin at a rate of 15 pmol kg^−1^ min^−1^ to increase plasma insulin within a physiological range. Blood samples were collected at 10~20 min intervals for measurements of plasma glucose, and 20% glucose was infused at variable rates to maintain baseline glucose levels. Insulin-stimulated whole-body glucose metabolism was estimated with a continuous infusion of [^3^H]glucose throughout the clamps (3700 Bq/min). At the end of the clamp study, we injected 2-[1-14C] deoxy-D-glucose (2-[^14^C]DG) to determine tissue-specific insulin-stimulated glucose uptake. 2-[^14^C]DG was administered as a bolus (370000 Bq) at 75 min after the start of clamp. Blood was sampled at 80, 85, 90, 100, 110 and 120 min of the clamp for the measurement of plasma [^3^H]glucose, ^3^H_2_O, and 2-[^14^C]DG concentrations.

Whole-body glucose turnover was measured as the ratio of the [^3^H]glucose infusion rate to the specific activity of plasma glucose at the end of the basal period. Insulin-stimulated whole-body glucose uptake was determined as the ratio of the [^3^H]glucose infusion rate to the specific activity of plasma glucose during the final 30 min of clamps. Hepatic glucose production during the clamp procedure was determined by subtracting the glucose infusion rate from the whole-body glucose uptake. Hepatic insulin sensitivity was calculated by dividing clamp hepatic glucose production by baseline glucose production and subtracting that number from 100 before presenting the results in percentage as described previously [25].

For experiments involving weight-matched (non-obese) mice, all the measurements were performed within 6 to 8 weeks of inducing *Adgrl1*^VMH^-deficiency, during which time the body weight was not different between the two groups of mice.

### Electrophysiology

To determine whether ADGRL1-expressing neurons are glucose sensitive, we generated an *Adgrl1*^Cre^ mouse model (Taconic/Cyagen, USA). An IRES-Cre cassette was inserted into the 3′ UTR region (70 bp after the TGA stop codon). To engineer the targeting vector, homology arms were generated by PCR using bacterial artificial chromosome (BAC) clone RP23-162K1 as a template. Cas9 and gRNA were co-injected into fertilised eggs with the targeting vector to generate the mice. The pups were genotyped by PCR followed by sequencing analysis. F1 mice were bred with WT C57BL/6J mice for at least six generations to obtain heterozygotes followed by production of homozygous mice on the C57BL/6J background. The following primers were used for genotyping the mice: 5′-ACAGGGCTACTACCAGGTGC-3′ and 5′-CATTCAACAGACCTTGCATTCCTTT-3′; 5′-ACAGGGCTACTACCAGGTGC-3′ and 5′-CTGGGCTTTCTCGTGGTATAAGG-3′. A band at 386 bp indicated the mutant allele and at 630 bp indicated the WT allele. For electrophysiological recordings, *Adgrl1*^Cre^ mice were injected with AAV2-FLEX-tdTomato virus (500 nl, 7×10^12^ vg/ml, 28306-AAV2, Addgene, USA) into the VMH as described above to label *Adgrl1*-expressing neurons with red fluorescence.

Mice were deeply anaesthetised with isoflurane and transcardially perfused with cold artificial cerebral spinal fluid (aCSF) (124 mmol/l NaCl, 4.5 mmol/l KCl, 1 mmol/l MgCl_2_, 26 mmol/l NaHCO_3_, 1.2 mmol/l NaH_2_PO_4_, 10 mmol/l glucose, 2 mmol/l CaCl_2_) bubbled with 95% O_2_ and 5% CO_2_. The brain was removed and submerged in an ice-cold sucrose-based cutting solution (30 mmol/l NaCl, 4.5 mmol/l KCl, 1 mmol/l MgCl_2_, 26 mmol/l NaHCO_3_, 1.2 mmol/l NaH_2_PO_4_, 10 mmol/l glucose, 194 mmol/l sucrose). Coronal slices (250 μm) containing the VMH were cut using a Leica VT1200S vibratome, USA. The slices were stored for 60 min in oxygenated 2.5 mmol/l glucose aCSF (124 mmol/l NaCl, 4.5 mmol/l KCl, 1 mmol/l MgCl_2_, 26 mmol/l NaHCO_3_, 1.2 mmol/l NaH_2_PO_4_, 2.5 mmol/l glucose, 2 mmol/l CaCl_2_, pH 7.4) at 32°C, and then kept at room temperature prior to recording. The recordings were performed at 32°C in a chamber that was perfused with the same 2.5 mmol/l glucose oxygenated aCSF at a rate of about 2 ml/min. Fluorescently labelled VMH neurons were visualised with epifluorescence on an upright Olympus BX51WI microscope. Patch pipettes (3–5 MΩ) were filled with an internal solution containing 126 mmol/l K-gluconate, 4 mmol/l KCl, 10 mmol/l HEPES, 0.3 mmol/l Na-GTP, 4 mmol/l Mg-ATP and 10 mmol/l phosphocreatine (7.3 pH, 290–310 mOsm). Membrane potential recordings were performed in a whole-cell current clamp configuration. Spontaneous excitatory postsynaptic currents were recorded in a whole-cell voltage clamp configuration at −60 mV holding potential. Cells were discarded if the action potentials failed to cross 0 mV or if the access resistance changed more than 20%. During the recordings the slices were alternately exposed to one of either two different glucose testing concentrations (10 mmol/l and 0.2 mmol/l) for 10 min, and then back to the control 2.5 mmol/l glucose aCSF before using the other glucose test treatment. Recordings were made using a MultiClamp 700B amplifier, sampled at 50kHz with a Digidata 1550B using Clampex software (Molecular Devices). Data were analysed with pCLAMP 11.1 (Molecular Devices, USA) and Prism 8.0.1 (GraphPad, USA).

### Statistical analyses

Data are shown as mean ± SEM. Results were analysed by two-tailed Student’s unpaired or paired *t* test, one-way ANOVA or two-way ANOVA followed by a Bonferroni post hoc multiple comparison test when appropriate. A χ^2^ test was used for electrophysiological analysis of the categorical data obtained from glucose-responsive and non-responsive neurons. All analyses were performed using Prism version 8.0.1 (GraphPad, USA) and differences were considered statistically significant at *p*<0.05.

## Results

### ADGRL1 binds with glucose

**Fig. 1:**
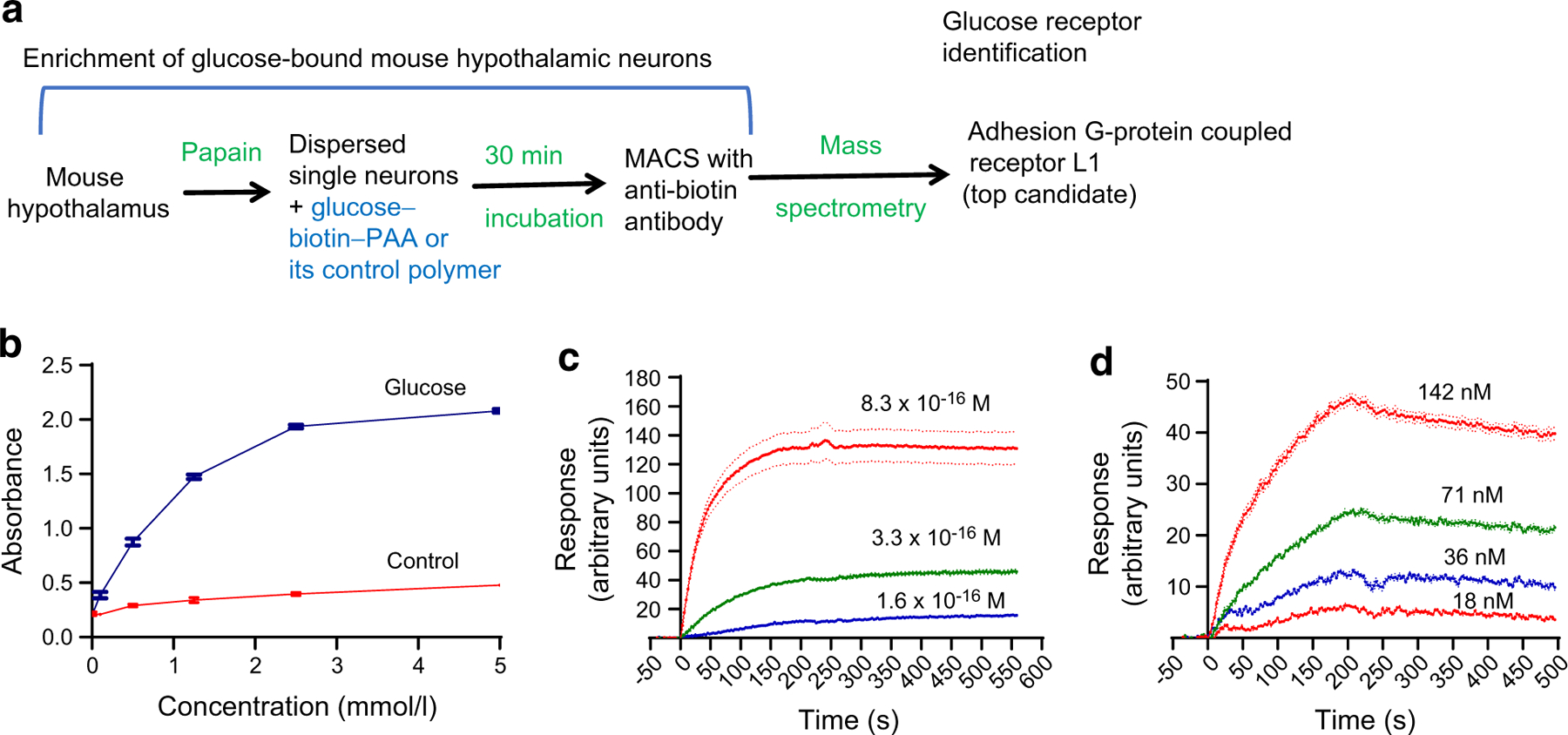
Glucose binds with ADGRL1. (**a**) Flow chart displaying the steps used in this study to identify a hypothalamic glucoreceptor. (**b**) Colorimetric assay showing dose-dependent binding of biotinylated glucose with human ADGRL1 stably expressed in CHO cells, *n*=4. (**c**, **d**) Glucose–ADGRL1 dose-dependent binding response obtained using SPRi, in which different concentrations of ADGRL1-expressing CHO cells were injected over immobilised biotinylated glucose (**c**), or different concentrations of soluble biotinylated glucose were injected over immobilised cells (**d**), *n*=3. M, mol/l of CHO cells stably expressing ADGRL1. nM, concentration of biotinylated glucose in nmol/l. K_D_ was 3.44×10^−14^ mol/l with the approach described in (**c**) and 4.86×10^−9^ mol/l with the method described in (**d**), both demonstrating that glucose binds ADGRL1. Ctrl, control

We used a 30 KDa glucose–biotin–PAA conjugate (ESM Fig. 1) and MACS technique to enrich glucose-bound hypothalamic neurons obtained from C57BL/6J mice. The enriched neurons were then lysed and subjected to proteomics analyses (Fig. 1a) to identify the most enriched receptors. We observed that ADGRL1 (encoded by *ADGRL1*, also known as *LPHN1*) was at the top of the list of potential glucose-binding proteins (ESM Tables 1 and 2).

To verify glucose–ADGRL1 binding, we produced a stable CHO-K1 cell line expressing human ADGRL1 (Uniprot O94910) and incubated the cells with different concentrations of glucose–biotin–PAA or its control conjugate (without glucose). We performed qRT-PCR to validate stable expression of ADGRL1 in CHO-K1 cells (2^−ΔΔC^t = 10,485, *ADGRL1*-expressing cells vs 1.0, control cells, *n*=3, fold relative to control). We then used biotin–streptavidin chemistry to detect the interaction between the conjugates and cells. The cells incubated with the glucose–biotin–PAA displayed a dose-dependent increase in absorbance, which was absent in the control group (Fig. 1b), thereby unequivocally demonstrating the binding of glucose with human ADGRL1. After validating glucose–ADGRL1 binding, we measured K_D_ to quantify binding affinity between the human ADGRL1-expressing cells and the biotinylated glucose or its control conjugate. When the conjugates were used as ligand (immobilised on a biochip) and the cells as analyte (injected over the conjugates), their interaction yielded K_on_ 3.5×10^8^ [mol/l]^−1^ s^−1^, K_off_ 1.24×10^−5^ s^−1^, and K_D_ 3.44×10^−14^ mol/l (Fig. 1c). In this approach, the cells are presented in a suspension form, hence they offer more binding surface area and may interact with several glucose molecules at the same time, thereby overestimating the binding affinity. To address this limitation, we also immobilised the cells (5×10^5^ cells/ml) on a biochip and then injected different concentrations of soluble glucose conjugates over them. This alternative approach yielded K_on_ 9.33×10^4^ [mol/l]^−1^ s^−1^, K_off_ 4.54×10^−4^ s^−1^, and K_D_ 4.86×10^−9^ mol/l (Fig. 1d), which may represent a more realistic measurement because only a portion of the cells was available to bind glucose, simulating a natural in vivo interaction. The cells without ADGRL1 yielded a negligible response with the glucose conjugate (ESM Fig. 2a). For the immobilisation procedure, we used a contact-spotting approach (described in the Methods) that demonstrated more optimal conditions and more consistent results than the microfluidic approach (ESM Fig. 2b, c). The use of the cells enabled us to study glucose–ADGRL1 interactions in situ, which presented the receptor in a form closest to its native state.

To account for the possibility that the glucose polymer, used in our initial studies to identify ADGRL1 as a glucose receptor, may not reflect the physiological properties of regular glucose, we further validated glucose–ADGRL1 binding using different concentrations of regular glucose (non-conjugated) immobilised onto 96 well plates and then adding recombinant human ADGRL1 protein solution into the wells to assess glucose–ADGRL1 interaction. Gel electrophoresis and colorimetry confirmed that glucose binds with recombinant ADGRL1 (ESM Fig. 3a, b).

We also determined the potential signal transduction pathway of the glucose–ADGRL1 interaction by measuring second messengers such as cAMP or Ca^2+^ in the stable CHO cells expressing ADGRL1 or its control in the presence or absence of glucose. We observed that glucose dose-dependently decreased cAMP (ESM Fig. 3c) in an assay used to detect coupling with Gαi protein. There was no change in cAMP or Ca^2+^ in assays used to detect couplings with Gs or Gq (ESM Fig. 3d, e).

### Global or hypothalamic ADGRL1 deficiency causes obesity in mice

**Fig. 2:**
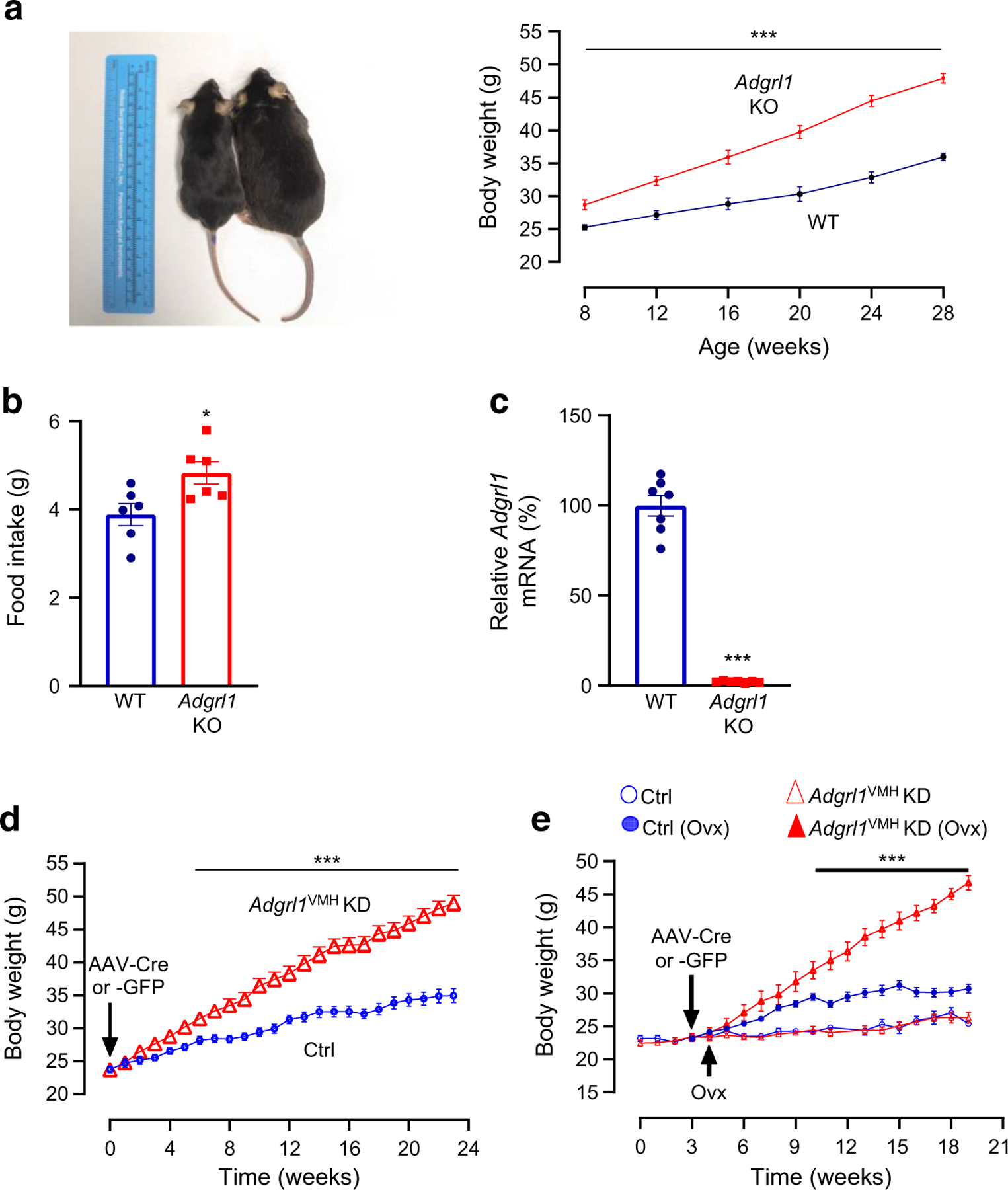
*Adgrl1*-deficient mice develop obesity. (**a**, **b**) Male global *Adgrl1* KO mice have higher body weight (**a**) and exhibit hyperphagia (**b**) (at 12 weeks of age). (**c**) *Adgrl1* gene KO was validated by qRT-PCR in the hypothalamus. *Adgrl1* KO mice and their littermate WT mice were euthanised at 29 weeks of age for qRT-PCR. (**d**, **e**) *Adgrl1* knockdown in the VMH (*Adgrl1*^VMH^ KD) was induced in 8-week-old male and female mice. (**d**) Body weight in male *Adgrl1*^VMH^ KD mice and littermate controls was measured at different times; *n*=15 Ctrl and *n*=25 *Adgrl1*^VMH^ KD mice. (**e**) Body weight in female *Adgrl1*^VMH^ KD mice and their littermate controls; ovariectomy in corresponding groups was performed 1 week after inducing the *Adgrl1* deficiency; *n*=10 Ctrl and *n*=8 *Adgrl1*^VMH^ KD mice. Data are shown as means ± SEM. Two-tailed Student’s unpaired *t* test or repeated measures ANOVA (1- or 2-way) followed by Bonferroni multiple comparison test: *p<0.05, ***p<0.001 vs Ctrl, over the indicated time points. Ctrl, control; Ovx, ovariectomy/ovariectomised mice

To determine the physiological role of ADGRL1 in energy balance, we produced global *Adgrl1* KO mice and measured their body weight and food intake. The *Adgrl1* KO mice exhibited obesity and hyperphagia was observed when they were about 12 weeks of age (Fig. 2a,b). We validated the knockout of *Adgrl1* using RT-qPCR (Fig. 2c). Because the hypothalamus is a major hub controlling body weight, feeding and glucose homeostasis [23, 27–29] we next determined tissue-specific distribution of *Adgrl1* mRNA in the hypothalamus and defined the role of hypothalamic ADGRL1 in energy homeostasis in male and female mice as described below.

We used an RNA in situ hybridisation procedure to assess the distribution of *Adgrl1* in the mouse hypothalamus. We report that *Adgrl1* is highly expressed in the VMH (ESM Fig. 4a, b) relative to other hypothalamic regions such as the paraventricular (PVH) and arcuate nucleus (ARC) of the hypothalamus. *Adgrl1* is colocalised with *Sf1*, which encodes steroidogenic factor 1 (SF1), a VMH marker (ESM Fig. 4c). Further, we observed that *Adgrl1* was mainly expressed in neurons as determined by the neuronal marker neuronal nuclear protein (*NeuN*; also known as *Rbfox3*; ESM Fig. 4d). We analysed *Adgrl1*–*NeuN* co-localisation using CellProfiler analysis software. About 94% of *Adgrl1*-expressing cells in the VMH showed *NeuN* staining (three sections/mouse brain and four mice/group were analysed).

To knock down *Adgrl1* specifically in the VMH (*Adgrl1*^VMH^), we generated *Adgrl1*^loxP/loxP^ mice (ESM Fig. 5a, b) and injected AAV-Cre or its control vector into the VMH of these mice using a stereotaxic surgical procedure (ESM Fig. 5c). The ADGRL1-deficient mice had higher body weight about 9 weeks after inducing ADGRL1 deficiency and gained ~47% more weight compared with their littermate controls by the 24th week following the *Adgrl1* knockdown (Fig. 2d). Given the involvement of the VMH in sex-dependent regulation of energy balance [30, 31], we determined whether ADGRL1 deficiency in the VMH produced obesity in female mice. We found that ADGRL1 deficiency alone did not cause obesity in female mice (Fig. 2e). To answer whether oestrogen is responsible for protecting the ADGRL1-deficient mice from obesity, we performed ovariectomy in these mice and their littermate controls. Indeed, the ovariectomised ADGRL1-deficient mice had higher body weight relative to their control ovariectomised mice (Fig. 2e), thereby unmasking the contribution of ADGRL1 in body weight regulation. After completion of the study, we validated the lack of ADGRL1 selectively in the VMH (ESM Fig. 5d) using RNA in situ hybridisation.

*Adgrl1*^VMH^-knockdown mice exhibited higher food intake (ESM Fig. 6a) on the 21st week after inducing the ADGRL1 deficiency, at which time the mice were already obese. We then used proton magnetic resonance spectroscopy (1H-MRS) to measure their body composition. *Adgrl1*^VMH^-deficient mice had higher fat and lean mass relative to their littermate controls (ESM Fig. 6b, c). Moreover, physical activity was reduced (ESM Fig. 6d) in the ADGRL1-deficient mice compared with their littermate controls. To determine whether the decrease in physical activity contributed to the development of obesity or was a consequence of obesity, we measured physical activity in *Adgrl1*^VMH^-deficient mice on the 6th week after inducing *Adgrl1*^VMH^ deficiency, during which time the mice displayed normal body weight (ESM Fig. 6e). The non-obese *Adgrl1*^VMH^-deficient mice were also less active than their littermate controls (ESM Fig. 6f). These findings suggest that the ADGRL1 deficiency is likely responsible for reducing physical activity in mice before they develop obesity. Moreover, the obese and weight-matched ADGRL1-deficient mice had normal energy expenditure when adjusted by total body mass using the MMPC ANCOVA analysis, but lower respiratory exchange ratio compared with their littermate controls (ESM Fig. 7a–d), suggesting elevated lipid oxidation in the ADGRL1-deficient mice.

### ADGRL1 in the VMH is involved in feeding responses to glucose or fasting

**Fig. 3:**
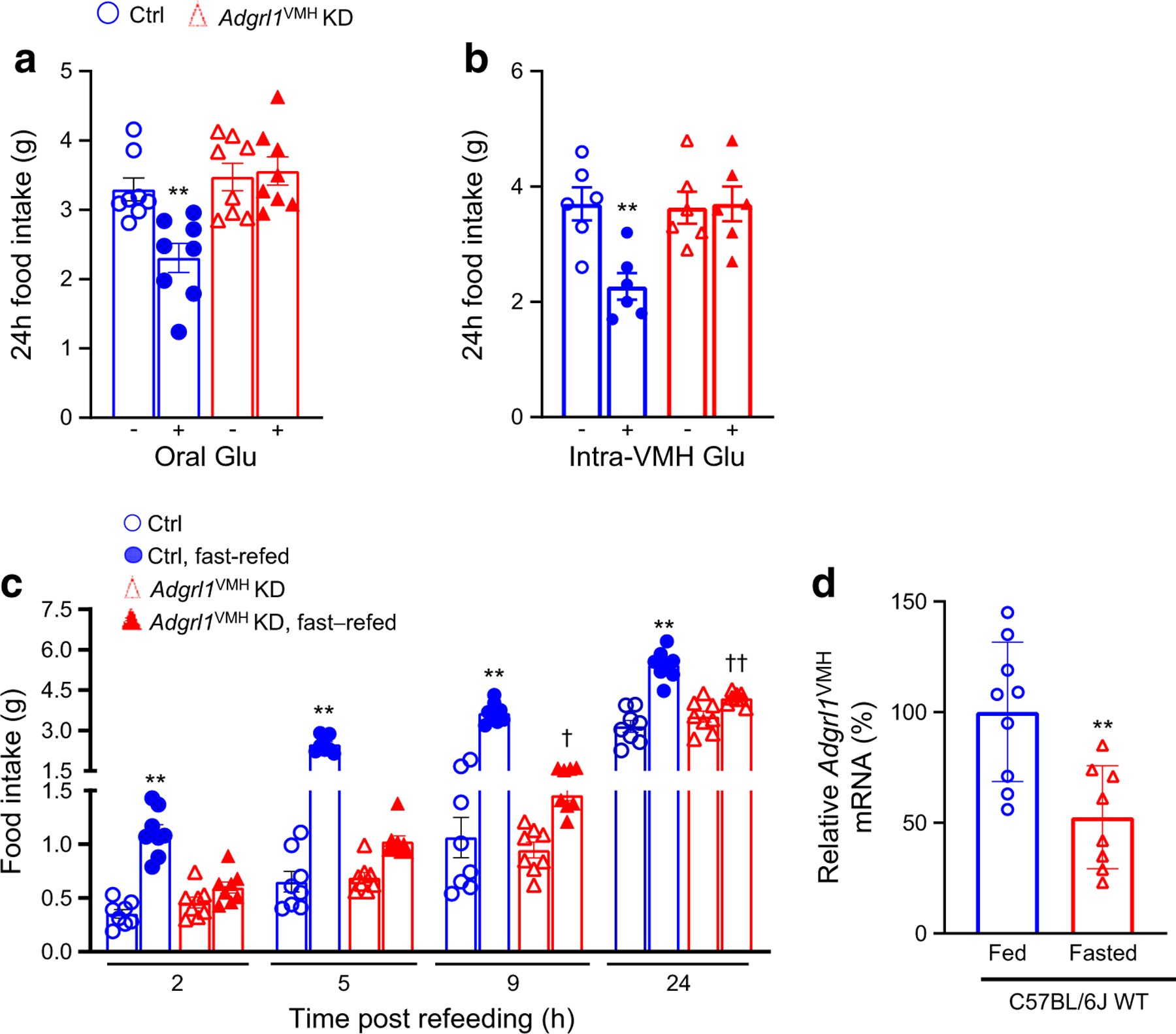
Impaired feeding responses in male *Adgrl1*^VMH^ KD (knockdown of *Adgrl1* in the VMH) mice and effect of fasting on *Adgrl1*^VMH^ expression. (**a**, **b**) Feeding responses to oral (**a**) or intra-VMH (**b**) glucose administration (500 mg glucose in 300 μl water, oral gavage, or 5 mmol/l glucose in 2 μl PBS, intra-VMH, administered at 16:00 and 17:00 hours for both) in the 3rd week following *Adgrl1*^VMH^ deficiency in 11-week-old *Adgrl1*^VMH^ KD mice and their littermate controls; *n*=6 or 8 Ctrl and *Adgrl1*^VMH^ KD mice. Two-tailed Student’s *t* test: **p<0.01 vs corresponding group without glucose. (**c**) Feeding response following overnight (18:00 to 09:00 hours) fasting in the 3rd week after *Adgrl1*^VMH^ deficiency in 11-week-old *Adgrl1*^VMH^ KD mice and their littermate controls; *n*=8 Ctrl and *Adgrl1*^VMH^ KD mice. (**d**) Overnight (18:00 to 09:00 hours) fasting reduces *Adgrl1* mRNA levels (measured by qRT-PCR) in the VMH of 8-week-old C57BL/6 male mice. Two-tailed Student’s unpaired *t* test or repeated measures two-way ANOVA followed by Bonferroni multiple comparison tests: **p<0.01 vs all other groups at corresponding times. ^†^*p*<0.05, ^††^*p*<0.01 vs *Adgrl1*^VMH^ KD (non-fasted) at the corresponding time. Ctrl, control; Glu, glucose

Glucose influences food intake [2–5]. According to the glucostatic theory and previous publications [2–5, 7, 8, 21], food intake is inversely proportional to blood glucose levels under normal circumstances. To determine the role of ADGRL1 in regulating glucose-mediated changes in food intake, we measured feeding responses to glucose administration or overnight fasting in non-obese *Adgrl1*^VMH^-deficient mice 3 weeks after inducing ADGRL1 deficiency using AAV-Cre as described above. Based on previous studies using this viral vector approach, 3 weeks are sufficient for mice to recover from stereotaxic surgery and for knockdown of desired genes [32]. The ADGRL1-deficient mice had impaired feeding responses compared with their littermate controls. The control mice did reduce their food intake, as anticipated, following oral or intra-VMH glucose administration, but lack of ADGRL1 in the VMH suppressed these responses (Fig. 3a,b). Conversely, we observed a temporary hyperphagia, as expected, in the control mice when they were refed ad libitum food following an overnight fast (Fig. 3c). However, this response was attenuated in ADGRL1-deficient mice (Fig. 3c). To clarify whether the levels of ADGRL1 are regulated by fasting, we used qRT-PCR to measure *Adgrl1* gene expression in the VMH of mice that were fasted overnight. We observed that the fasting downregulated the *Adgrl1* expression (Fig. 3d), further suggesting the role of ADGRL1 in responding to changes in energy balance. These results demonstrate the contribution of ADGRL1 in the VMH to mediating feeding responses to glucose and fasting in mice.

### ADGRL1 in the VMH regulates insulin secretion and insulin sensitivity

**Fig. 4:**
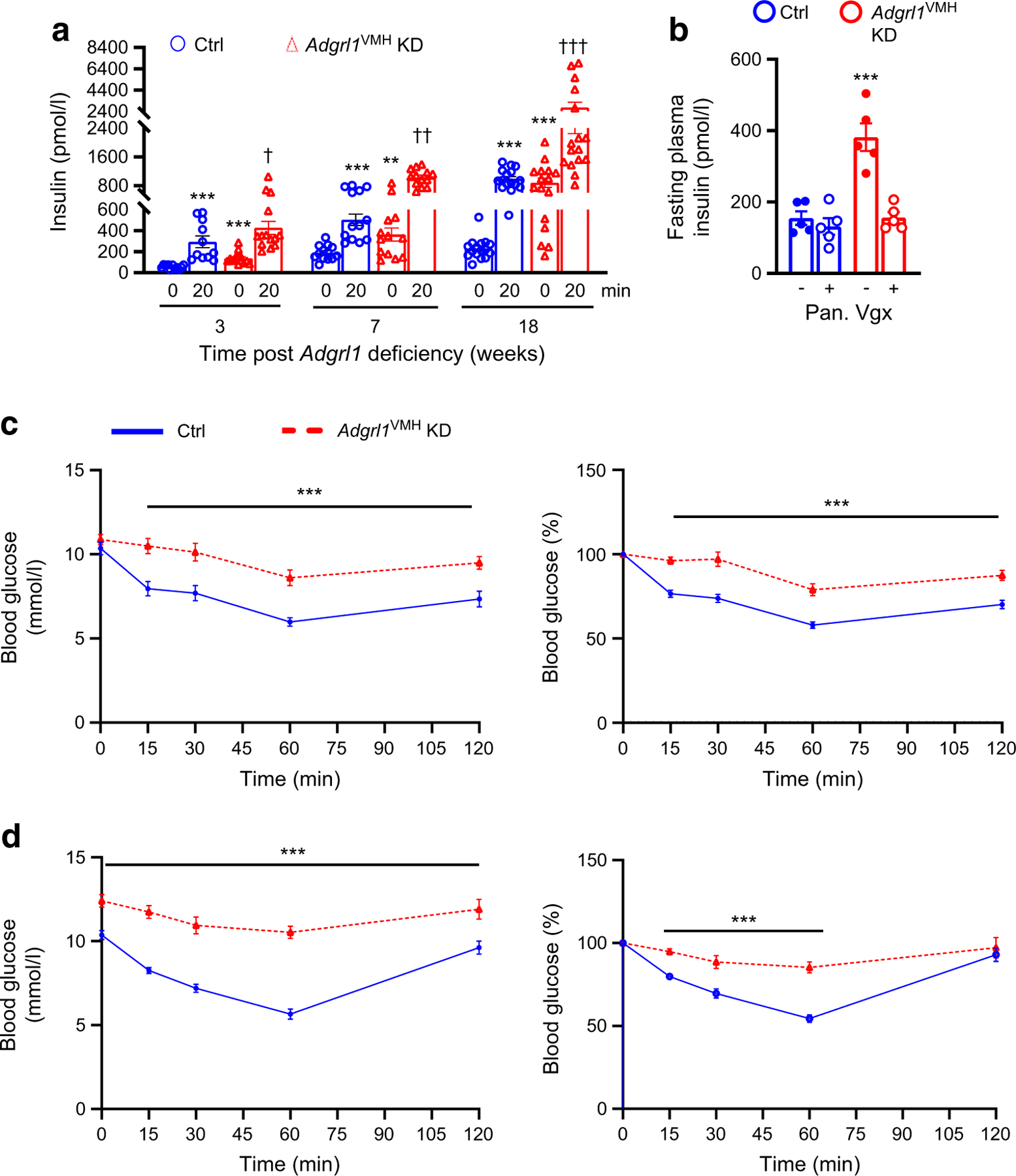
Hyperinsulinaemia in male *Adgrl1*^VMH^ KD (knockdown of *Adgrl1* in the VMH) mice. (**a**) Fasting (08:00 to 14:00 hours) plasma insulin levels (0 min) and glucose-stimulated insulin secretion (20 min). The weeks on the *x*-axis indicate the time after inducing *Adgrl1*^VMH^ deficiency; *n*=11 Ctrl and *n*=14 *Adgrl1*^VMH^ KD (3 weeks following *Adgrl1*^VMH^ deficiency), *n*=13 Ctrl and *Adgrl1*^VMH^ KD (7 weeks following *Adgrl1*^VMH^ deficiency), *n*=16 Ctrl and *Adgrl1*^VMH^ KD (18 weeks following *Adgrl1*^VMH^ deficiency). Repeated measures two-way ANOVA followed by Bonferroni multiple comparison tests: **p<0.01, ***p<0.001 vs Ctrl at 0 min (for the corresponding time post *Adgrl1* deficiency). ^†^*p*<0.05, ^††^*p*<0.01, ^†††^*p*<0.001 vs all other groups at corresponding times. (**b**) Pancreatic vagotomy reverses hyperinsulinaemia in *Adgrl1*^VMH^ KD mice within 3 days of the vagotomy. Vagotomy was performed 4 weeks after inducing the *Adgrl1* deficiency. Two-way ANOVA followed by Bonferroni multiple comparison test: ***p<0.001 vs all other groups. (**c**, **d**) Impaired insulin sensitivity in *Adgrl1*^VMH^ KD mice. Insulin tolerance tests (raw data and presented as % of baseline blood glucose level) on the 15th week (**c**) and 24th week (**d**) after inducing *Adgrl1* deficiency; *n*=12 Ctrl and *n*=10 *Adgrl1*^VMH^ KD mice in (**c**), *n*=16 Ctrl and *Adgrl1*^VMH^ KD mice in (**d**). Two-way repeated measures ANOVA followed by Bonferroni multiple comparison tests: ***p<0.001. Ctrl, control; Pan. Vgx, pancreatic vagotomy

VMH lesions cause hypersecretion of insulin [33, 34]. Because ADGRL1 is highly expressed in the VMH, we measured fasting plasma insulin levels and glucose-stimulated insulin secretion in *Adgrl1*^VMH^-deficient mice to determine the involvement of VMH ADGRL1 in regulating insulin secretion. Compared with the control group, the ADGRL1-deficient mice had increased fasting plasma insulin levels at baseline as well as after glucose administration (Fig. 4a), thereby amplifying glucose-stimulated insulin secretion. As expected from previous studies [35, 36], the control mice showed a progressive increase in plasma insulin levels under fasting and glucose-stimulated conditions with age, which was augmented in *Adgrl1*^VMH^-deficient mice. The first analyses of fasting plasma insulin levels and glucose-stimulated insulin secretion were performed 3 weeks after inducing the ADGRL1 deficiency. At this time, the ADGRL1-deficient mice and their control group had similar body weights and insulin sensitivity (ADGRL1-deficient mice vs control group: body weight, 26.5 ±1 vs 25.1 ±0.6 g; AUC of blood glucose levels obtained during insulin tolerance test, 966 ± 83.34 vs 884.2 ± 71.48 mmol/l × min, *n*=11 and 14, respectively), indicating that the fasting hyperinsulinaemia and glucose-stimulated hypersecretion of insulin were consequences of ADGRL1 deficiency and not secondary to obesity or insulin resistance. Given the contribution of the vagus nerve to insulin secretion following hypothalamic lesions [33], we denervated the pancreatic vagal nerve to determine whether it mediates hyperinsulinaemia in the ADGRL1-deficient mice. Within three days of pancreatic vagotomy, the insulin hypersecretion was reversed (Fig. 4b) These findings imply that ADGRL1 in the VMH may be responsible for keeping a check on insulin secretion via the vagus nerve to maintain physiological levels of circulating insulin at baseline and following meals.

**Fig. 5:**
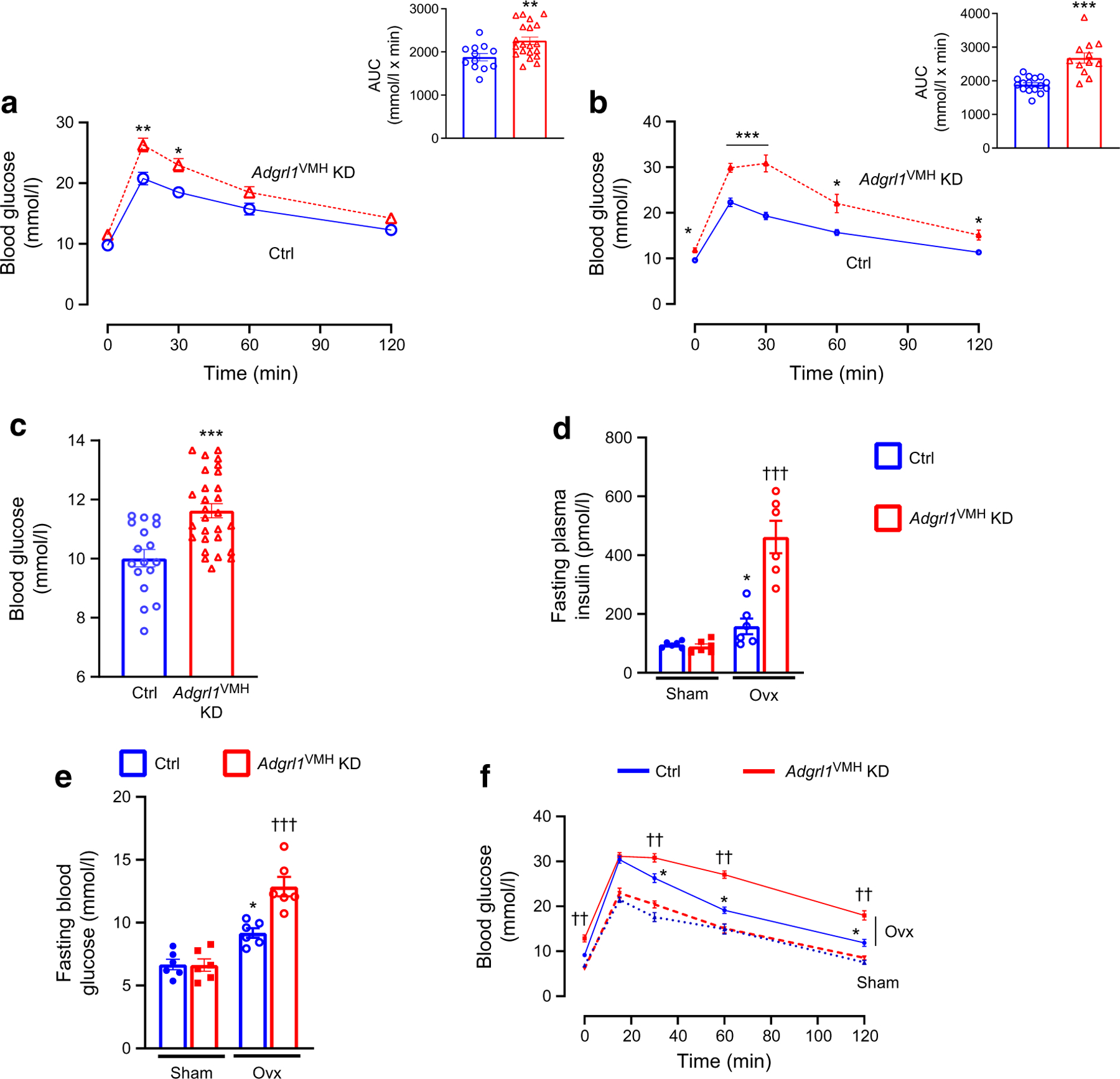
Impaired glucose homeostasis in *Adgrl1*^VMH^ KD (knockdown of *Adgrl1* in the VMH) male and ovariectomised female mice. (**a**, **b**) Oral glucose tolerance test in the 17th week (**a**; *n*=12 Ctrl and *n*=20 *Adgrl1*^VMH^ KD mice) and 24th week (**b**; *n*=16 Ctrl and *n*=12 *Adgrl1*^VMH^ KD male mice) after inducing *Adgrl1*^VMH^ deficiency. Bar graphs in (**a**) and (**b**) represent the corresponding AUC. (**c**) Fasting (08:00 to 14:00 hours) blood glucose levels on the 24th week following *Adgrl1* deficiency, *n*=17 Ctrl and *n*=28 *Adgrl1*^VMH^ KD male mice. (**d**, **e**, **f**) Ovariectomised *Adgrl1*^VMH^ knockdown (*Adgrl1*^VMH^ KD) female mice have higher plasma insulin (**d**), fasting hyperglycaemia (**e**) and impaired glucose tolerance (**f**) compared with their littermate controls. Ovariectomy or sham surgery was performed in 9-week-old mice 1 week after inducing *Adgrl1*^VMH^ deficiency. Plasma insulin was measured 3 weeks after inducing the *Adgrl1* deficiency. Fasting blood glucose levels and oral glucose tolerance were measured 18 weeks after inducing the *Adgrl1* deficiency. Data are shown as means ± SEM. Two-way repeated measures ANOVA followed by Bonferroni multiple comparison tests or two-tailed Student’s unpaired *t* test: *p<0.05, **p<0.01, ***p<0.001 vs Ctrl or sham, ^††^*p*<0.01, ^†††^*p*<0.001 vs all other groups. Ctrl, control; Ovx, ovariectomised mice

Next, we measured insulin sensitivity and glucose tolerance in *Adgrl1*^VMH^-deficient mice to further determine whether these mice show insulin resistance followed by impairments in glucose regulation. We observed that the *Adgrl1*^VMH^-deficient mice had reduced insulin sensitivity, as measured by insulin tolerance tests, compared with their littermate controls at different times throughout the study (Fig. 4c,d). The *Adgrl1*^VMH^-deficient mice exhibited impaired glucose tolerance on the 17th and 24th week after inducing the ADGRL1 deficiency (Fig. 5a,b). The impaired glucose tolerance was likely due to the chronic insulin resistance observed in the *Adgrl1*^VMH^-deficient mice (Fig. 4c,d). The ADGRL1-deficient mice exhibited fasting hyperglycaemia (Fig. 5c) on the 24th week following ADGRL1 knockdown. Similarly, the ovariectomised female *Adgrl1*^VMH^-deficient mice also manifested hyperinsulinaemia, hyperglycaemia and impaired glucose tolerance (Fig. 5d–f). As expected, ovariectomy itself induced metabolic abnormalities; however, these were exacerbated in the ADGRL1-deficient mice, thereby revealing the effects of ADGRL1 on energy and glucose homeostasis in female mice.

**Fig. 6:**
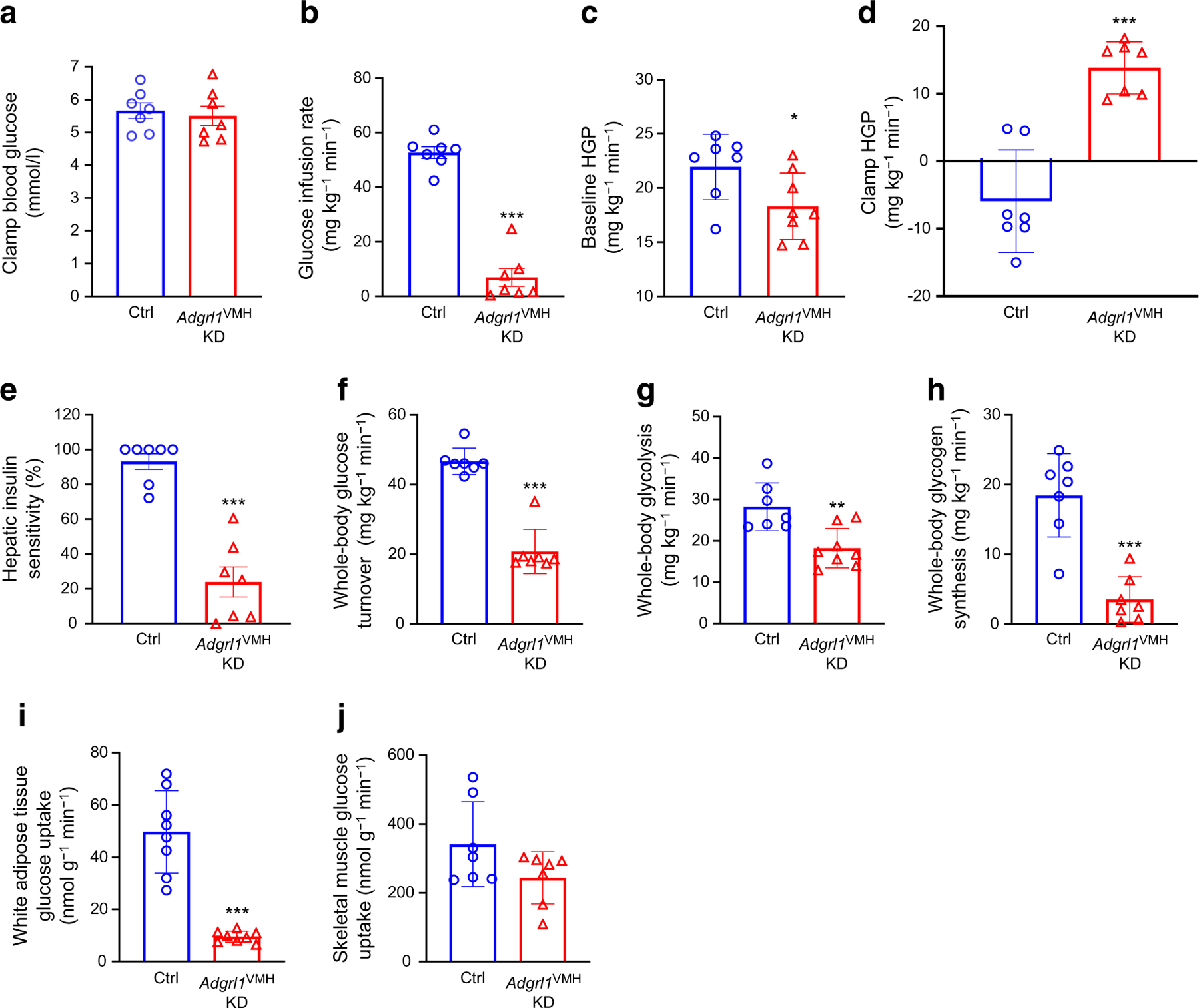
Hyperinsulinaemic–euglycaemic clamps in obese male *Adgrl1*^VMH^ KD (knockdown of *Adgrl1* in the VMH) mice. (**a**) Clamped blood glucose levels; (**b**) glucose infusion rate; (**c**) baseline and (**d**) clamp hepatic glucose production; (**e**) hepatic insulin sensitivity; (**f**) whole-body glucose turnover; (**g**) whole-body glycolysis; (**h**) whole-body glycogen synthesis; (**i**) glucose uptake in adipose tissue and (**j**) skeletal muscle during hyperinsulinaemic–euglycaemic clamps in the 22nd week following *Adgrl1*^VMH^ deficiency in 30-week-old male *Adgrl1*^VMH^ KD mice and their littermate controls; *n*=7 Ctrl and *Adgrl1*^VMH^ KD. Data are shown as mean ± SEM. Two-tailed Student’s *t* test: *p<0.05, **p<0.01, ***p<0.001. Ctrl, Control; HGP, hepatic glucose production

To further validate insulin resistance and impaired glucose homeostasis, we performed hyperinsulinaemic–euglycaemic clamps in awake *Adgrl1*^VMH^-deficient mice and their littermate controls (Fig. 6a). The ADGRL1-deficient mice needed less exogenous glucose to maintain their clamped blood glucose levels compared with the control mice (Fig. 6b), indicating insulin resistance in the ADGRL1-deficient mice. Moreover, the ADGRL1 deficiency led to impaired baseline glucose production and desensitized insulin-mediated suppression of hepatic glucose production (Fig. 6c–e). The *Adgrl1*^VMH^-deficient mice had defective glucose turnover, glycolysis and glycogen synthesis under the hyperinsulinaemic conditions (Fig. 6f–h), indicating impaired insulin sensitivity. The clamp procedure further revealed the sites of insulin resistance. The ADGRL1-deficient mice had decreased insulin-mediated glucose uptake in white adipose tissue (epididymal) but not gastrocnemius skeletal muscle (Fig. 6i,j). We repeated the hyperinsulinaemic–euglycaemic clamp study in the non-obese *Adgrl1*^VMH^-deficient mice (weights shown in ESM Fig. 6e), 6 weeks after inducing ADGRL1 deficiency in the VMH, when their weights were similar to that of the control littermates. The weight-matched *Adgrl1*^VMH^-deficient mice also manifested insulin resistance (ESM Fig. 8a–f) except in gastrocnemius skeletal muscle (ESM Fig. 8g), thereby supporting the findings obtained from the obese *Adgrl1*^VMH^-deficient mice. Together, these results demonstrate that VMH ADGRL1 is indispensable for maintaining physiological plasma insulin levels and insulin sensitivity to consequently influence glucose homeostasis.

### ADGRL1 contributes to glucose sensing

**Fig. 7:**
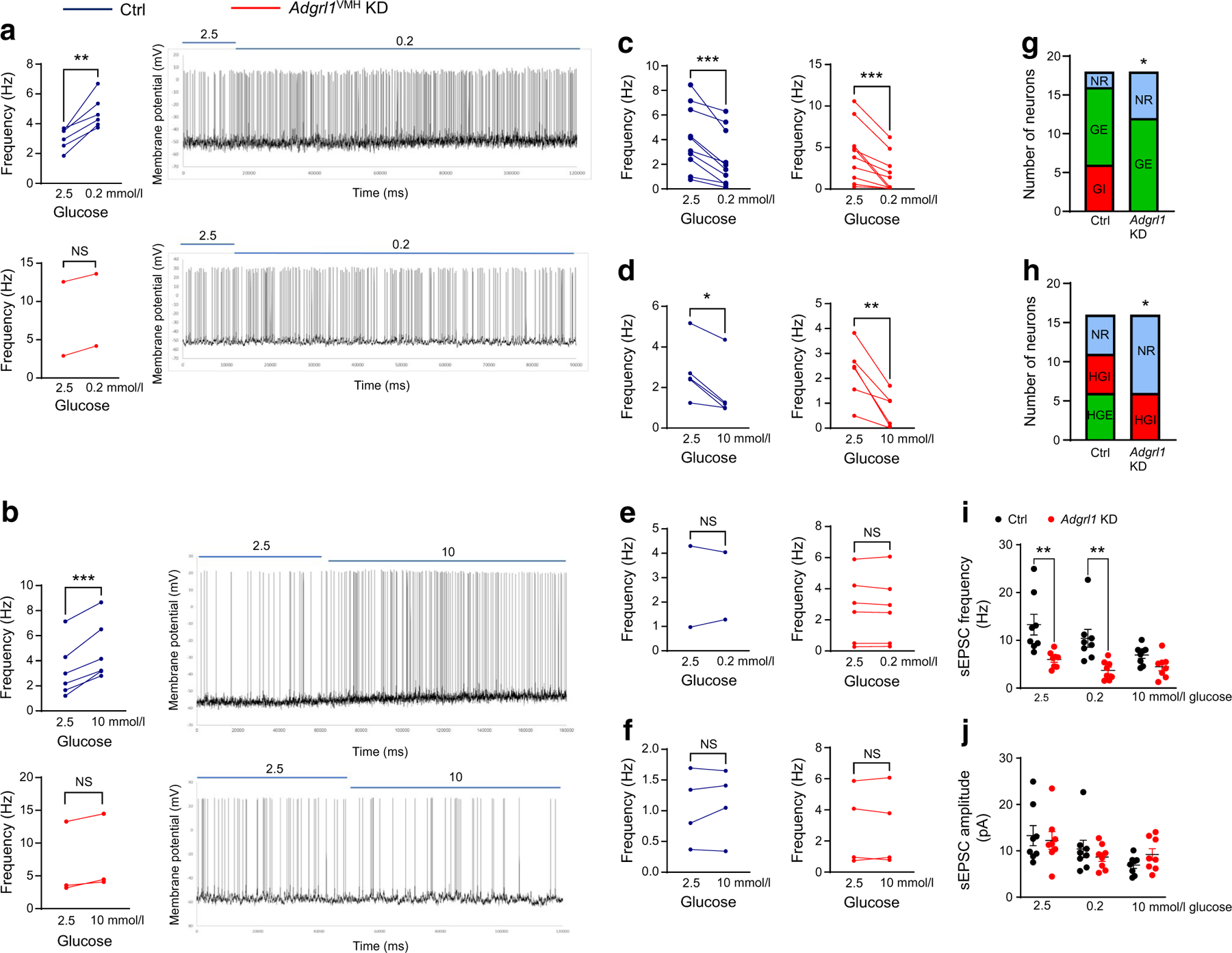
Electrophysiological characterisation of glucose-responsive neurons in the presence (control) and knockdown (*Adgrl1*^VMH^ KD) of *Adgrl1* in the VMH 6 weeks after inducing *Adgrl1* deficiency in 14-week-old non-obese male mice. Representative traces are shown for the categories that were different between the groups in their response to glucose. Six out of 34 recorded neurons showed an increase in action potential firing in response to change in glucose concentration from 2.5 to 0.2 mmol/l (GI neurons); only two neurons in the *Adgrl1*^VMH^ KD group showed such a trend and this was non-significant (**a**). Six out of 34 recorded neurons showed an increase in action potential firing in response to change in glucose concentration from 2.5 to 10 mmol/l (HGE neurons); only three neurons in the *Adgrl1*^VMH^ KD group showed such a trend and this was non-significant (**b**). (**c**, **d**) The number of GE neurons (**c**) and HGI neurons (**d**) was similar between the groups. (**e**, **f**) The remaining recorded neurons were not responsive to low (**e**) or high (**f**) glucose. (**g**, **h**) Summary of the proportion of changes in glucose-sensing neurons. (**i**, **j**) The frequency of spontaneous excitatory postsynaptic currents was reduced in the *Adgrl1*^VMH^ KD mice at 2.5 and 0.2 mmol/l glucose (**i**), but there was no change in the mean amplitude of sEPSCs at different glucose concentrations (**j**). Data are presented from individual recorded neurons. Two or three neurons per brain slice were recorded and two or three slices per mouse (five mice per group) were included in this study. Two-tailed Student’s paired *t* test or *χ*^2^ test: *p<0.05, **p<0.01, ***p<0.001. NR, not responsive; sEPSC, spontaneous excitatory postsynaptic current

We performed mouse brain slice electrophysiology to determine the role of VMH ADGRL1 in glucose sensing according to the criteria established in previous publications [19, 37]. ADGRL1-expressing and knocked-down neurons were identified in the VMH using a fluorescent microscope as described in the Methods. ADGRL1 neurons exhibited a heterogenous response to glucose and were classified as glucose-excited (GE), glucose-inhibited (GI), high-glucose-excited (HGE), and high-glucose-inhibited (HGI) neurons (Fig. 7), in line with previous publications [19, 37]. We did not observe any GI neurons (0 out of 34 neurons) in the VMH in the ADGRL1-deficient mice, while 18% of recorded neurons (6 out of 34 neurons) were GI in the control group (Fig. 7a,g). Similarly, we did not observe HGE neurons in the ADGRL1-deficient mice, compared with 18% HGE neurons (6 out of 34 neurons) observed in their control littermates (Fig. 7b,h). Lack of ADGRL1 reduced the proportion of glucose-sensing neurons mainly by reducing the number of GI and HGE neurons, thereby increasing the number of glucose non-responsive neurons (Fig. 7c–h). These data demonstrate a role for ADGRL1 in mediating the effects of glucose on neuronal excitability. In addition, the frequency of spontaneous excitatory postsynaptic currents was decreased with 0.2 and 2.5 mmol/l glucose, without affecting the amplitude, in ADGRL1-deficient neurons (Fig. 7i,j). This finding suggests that ADGRL1 plays a role in the ability of glucose to alter glutamate release at VMH synapses, but not on postsynaptic glutamate receptor responses. Altogether, these data implicate ADGRL1 as a crucial component in determining cellular excitability responses to changes in glucose.

## Discussion

### Statement of principal findings

We have identified ADGRL1 as a glucose receptor involved in regulating energy and glucose homeostasis. ADGRL1 deficiency in the hypothalamus causes insulin resistance, obesity and impairs glucose sensing in mice.

### Strengths and weaknesses of the study

ADGRL1 is a member of the adhesion G-protein coupled receptor family. We have validated glucose–ADGRL1 binding through different assays including an approach using normal concentration of regular non-conjugated glucose to demonstrate physiological relevance of our findings. It is important to note that K_D_ values reported in this study are approximate and will change depending on the methods used to measure them. We used the high molecular mass glucose conjugate to study glucose–ADGRL1 interactions. This highly sensitive glucose conjugate may have overestimated the affinity between glucose and ADGRL1. Nevertheless, both the SPRi approaches used in the present study demonstrate that human ADGRL1 binds glucose. In addition, the sequence analyses of mouse ADGRL1 (UniProt Q80TR1) show that it contains a lectin-like domain (glucose/carbohydrate binding domain). The structure of the lectin-like domain has been described previously [38] and Adgrl1 is also included in the UniLectin3D database (2JX9 and 2JXA, https://unilectin.unige.ch/unilectin3D/). Hitherto, ADGRL1 has mainly been studied in the context of experimental neurotransmission in response to its putative agonist α-latrotoxin, which is a potent excitatory neurotoxin [39–43]. Through the present study, we provide evidence that ADGRL1 binds glucose and is essential for regulating energy and glucose homeostasis.

Using a stable CHO-K1 cell line expressing human ADGRL1, we show that glucose–ADGRL1 coupling activates the Gαi pathway. These findings support previous studies indicating the coupling of ADGRL1 with the Gαi signalling [44], although these downstream signalling pathways vary depending on splice events [45] or cell systems [46, 47] affecting the expression of ADGRL1. Currently, it is not feasible to perform such studies directly using primary hypothalamic neuron culture because of limitations in generating stable ADGRL1-expressing primary neurons. Nevertheless, the coupling of ADGRL1 with Gαi signalling supports our observations in mice that hypothalamic ADGRL1 is a negative regulator of insulin secretion probably via the autonomic nervous system. These findings further corroborate the general view that activation of Gαi signalling reduces insulin secretion [48, 49]. Previous in vitro studies using beta cells have indicated the involvement of α-latrotoxin, a putative ADGRL1 agonist, in insulin secretion via exocytosis [50]. Moreover, ADGRL3, which has a lectin-like domain similar to that of ADGRL1 and was also observed on our list of potential glucose-binding proteins (ESM Table 2), is also suggested to influence insulin secretion in beta cells in vitro [51]. In the present study, we demonstrate the role of hypothalamic ADGRL1 in regulating insulin secretion and sensitivity.

The findings reported in this study are reminiscent of that observed with SF1 deficiency or genetic manipulations in SF1 neurons in the VMH [52–54]. For example, hyperinsulinaemia, insulin resistance and reduced physical activity appear to have contributed to obesity in mice deficient in ADGRL1 in the VMH because hyperphagia was observed after the development of obesity in these mice. Similarly, fasting hyperglycaemia and impaired glucose tolerance were observed secondary to insulin resistance and obesity. SF1 neurons, together with adrenergic signalling in the VMH, contribute to the beneficial effects of physical activity [55, 56]. In the present study, we demonstrate that *Adgrl1* mRNA colocalises with *Sf1* in the VMH. Moreover, RNA-sequencing results from a previous report show that *Adgrl1* is present in hypothalamic proopiomelanocortin (POMC) and agouti related protein (AGRP)-expressing neurons [57], which are known energy-sensing neurons and are regulated by physical activity [58]. Therefore, hypothalamic ADGRL1 in these neurons may contribute to their function in regulating energy homeostasis. SF1, POMC and AGRP neurons also influence sympathetic tone [56, 59, 60], whose role in modulating insulin sensitivity and physical activity is well established. Here, we show that fasting hyperinsulinaemia in *Adgrl1*^VMH^-deficient mice is reversed by vagal denervation, raising the possibility that ADGRL1 might also regulate sympathetic tone because of the known interactions between the sympathetic and parasympathetic nervous systems [61]. Integrating the results from the previous studies mentioned above and the findings reported here, it is likely that activation of ADGRL1 neurons in the VMH will enhance insulin sensitivity and physical activity. Taken together, ADGRL1 in the VMH appears to be at the intersection of energy homeostasis, physical activity and insulin sensitivity.

VMH neurons are known to sense glucose and respond to changes in local or systemic glucose levels [37, 62–65]. The electrophysiological recordings in the present study demonstrate that ADGRL1 in the VMH is involved in direct glucose sensing, and some ADGRL1-expressing neurons are inhibited by, and some excited by, glucose. These findings support the current literature about the heterogeneity of the types of cells in the VMH and their direct involvement in glucose sensing. For example, VMH neurons expressing SF1 [63], melanocortin 3 receptor [62], or pituitary adenylate cyclase-activating peptide (PACAP) [66] sense glucose and/or respond to direct changes in blood glucose levels. It would be interesting to observe whether or to what extent ADGRL1 in the VMH contributes to the recently identified role of VMH^PACAP^ neurons [66] in directly sensing blood glucose levels.

Ovariectomy was necessary in female mice to unmask the effects of ADGRL1 on energy homeostasis. While ovariectomy also affects other sex hormones, the contribution of oestrogen to regulating energy balance is well established [31, 67]. Oestrogen receptor α is present in SF1 neurons [30, 31] and is also involved in regulating physical activity [30, 31, 68]. Given the *Adgrl1*/*Sf1* co-localisation and the contribution of ADGRL1 to energy homeostasis coupled with physical activity as demonstrated in the present study, it is likely that ADGRL1 signalling interacts with that of oestrogen in integrating metabolism, reproduction and physical activity. Collectively, these findings further reinforce the significance of the protective effects of oestrogen in metabolic disorders and why post-menopausal women are at a higher risk for developing the metabolic syndrome. Therefore, studying sex differences in preclinical models is imperative to establish tailored treatments for diabetes and obesity.

### Meaning of the study

We have demonstrated that ADGRL1 binds with glucose and is essential for regulating energy and glucose homeostasis in mice. In female mice, the contribution of ADGRL1 to influencing body weight is manifested after ovariectomy. A genome-wide scan suggests that the *ADGRL1* locus on chromosome 19 (19p13) is associated with human obesity [69]. Therefore, our findings from the ADGRL1-deficient mouse models provide proof-of-principle to establish the contribution of ADGRL1 to human obesity. Targeting ADGRL1 may introduce a new class of drugs for treatment of type 2 diabetes and obesity.

### Unanswered questions and future research

Given that ADGRL1 is involved in synaptic transmission [39–43], its own contribution—independently of glucose signalling—to the phenotype observed in *Adgrl1*^VMH^-deficient mice needs to be investigated. Whether hypothalamic ADGRL1 influences insulin secretion through changes in the islet mass or function remains unknown. It is likely ADGRL1 regulates islet function through the autonomic nervous system given the established role of the vagus nerve in influencing islet physiology [70, 71]. Future studies are required to address whether or how ADGRL1 function is regulated by glucose, insulin, leptin, and different diets in widely used mouse models of the metabolic syndrome such as *db/db*, *ob/ob* and diet-induced obesity models. *Adgrl1*^VMH^-deficient mice show reduced physical activity, high lean mass and normal insulin-mediated glucose uptake in the skeletal muscle, while the liver and adipose tissue exhibit insulin resistance. Additional mechanistic studies are required to explain these tissue-specific differential effects of ADGRL1 on glucose uptake. Similarly to *Adgrl1*^VMH^-deficient mice, mice lacking melanocortin 4 receptor or the *Pomc* gene also show high lean mass [72, 73] and hypothalamic melanocortin signalling is involved in differential regulation of glucose uptake [74]. Finally, whether ADGRL1 deficiency influences glucose transport and affects glucose metabolism and/or expression of glucose transporters remains unknown and requires further investigation.

## Supplementary Material

Supp. Data

Supp. Figures

## What is already known about this subject?

- The hypothalamus expresses glucose receptors that respond to changes in glucose levels and energy balance
- Although glucose transporters such as GLUT2 and SGLT2 are widely known to regulate glucose metabolism, the identity of glucose receptors involved in glucose signalling pathways remains unclear

## What is the key question?

- Which receptors in the hypothalamus bind glucose and are involved in energy and/or glucose homeostasis?

## What are the new findings?

- Glucose binds adhesion G-protein coupled receptor L1 (ADGRL1) and activates the Gai signalling pathway
- Hypothalamic ADGRL1 deficiency causes hyperinsulinaemia and obesity and impairs glucose sensing in mice
- In female mice, ovariectomy is necessary to reveal the contribution of ADGRLI to energy and glucose homeostasis

## How might this impact on clinical practice in the foreseeable future?

- Targeting ADGRL1 may introduce a new class of drugs for the treatment of type 2 diabetes and obesity

## Abbreviations

ADGRL1: Adhesion G-protein coupled receptor L1
AGRP: Agouti related protein
CHO: Chinese hamster ovary
FDR: False discovery rate
GE: Glucose-excited
GI: Glucose-inhibited
HBSS: Hanks’ Balanced Salt Solution
HGE: High-glucose-excited
HGI: High-glucose-inhibited
HRP: Horseradish peroxidase
K_ATP_: ATP-sensitive potassium channel
K_D_: Equilibrium dissociation constant
KO: Knockout
K_on_: Association rate constant
K_off_: Dissociation rate constant
MACS: Magnetic activated cell sorting
MMPC: Mouse Metabolic Phenotyping Centers
PAA: Polyacrylamide
PACAP: Pituitary adenylate cyclase-activating peptide
POMC: Proopiomelanocortin
qRT-PCR: Quantitative reverse transcription PCR
SF1: Steroidogenic factor 1
SPRi: Surface plasmon resonance imaging
VMH: Ventromedial nucleus of the hypothalamus
WT: Wild-type
