# Supplementary material for: ADGRL1 is a glucose receptor involved in mediating energy and glucose homeostasis
Source: Diabetologia. Author manuscript; Available in PMC 2024 Feb 5. (PMC10709246; doi:10.1007/s00125-023-06010-6)
Supplement: Supp. Figures [file NIHMS1936616-supplement-Supp__Figures.pdf]

ESM Figures

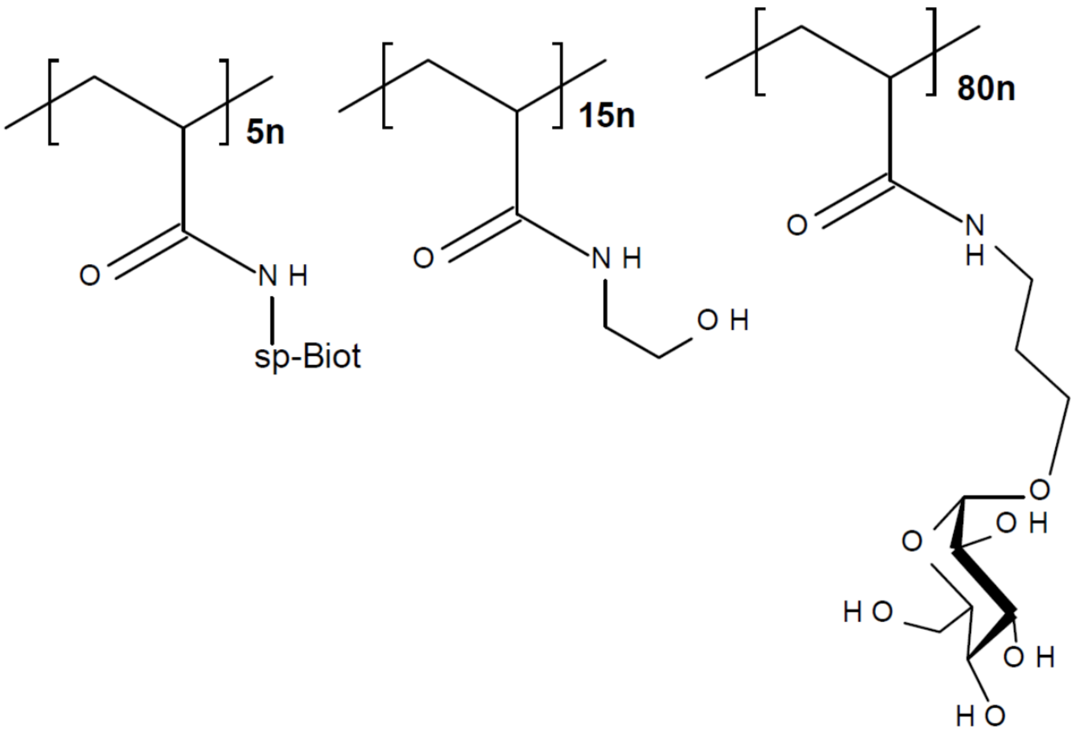

ESM Fig. 1 Structure of 30 KDa multivalent biotinylated glucose. Glucose is incorporated into a polyacrylamide matrix with 80 mol% glucose, 15 mol% polyacrylamide, and 5 mol% biotin. sp, spacer; n, number of units linked together.

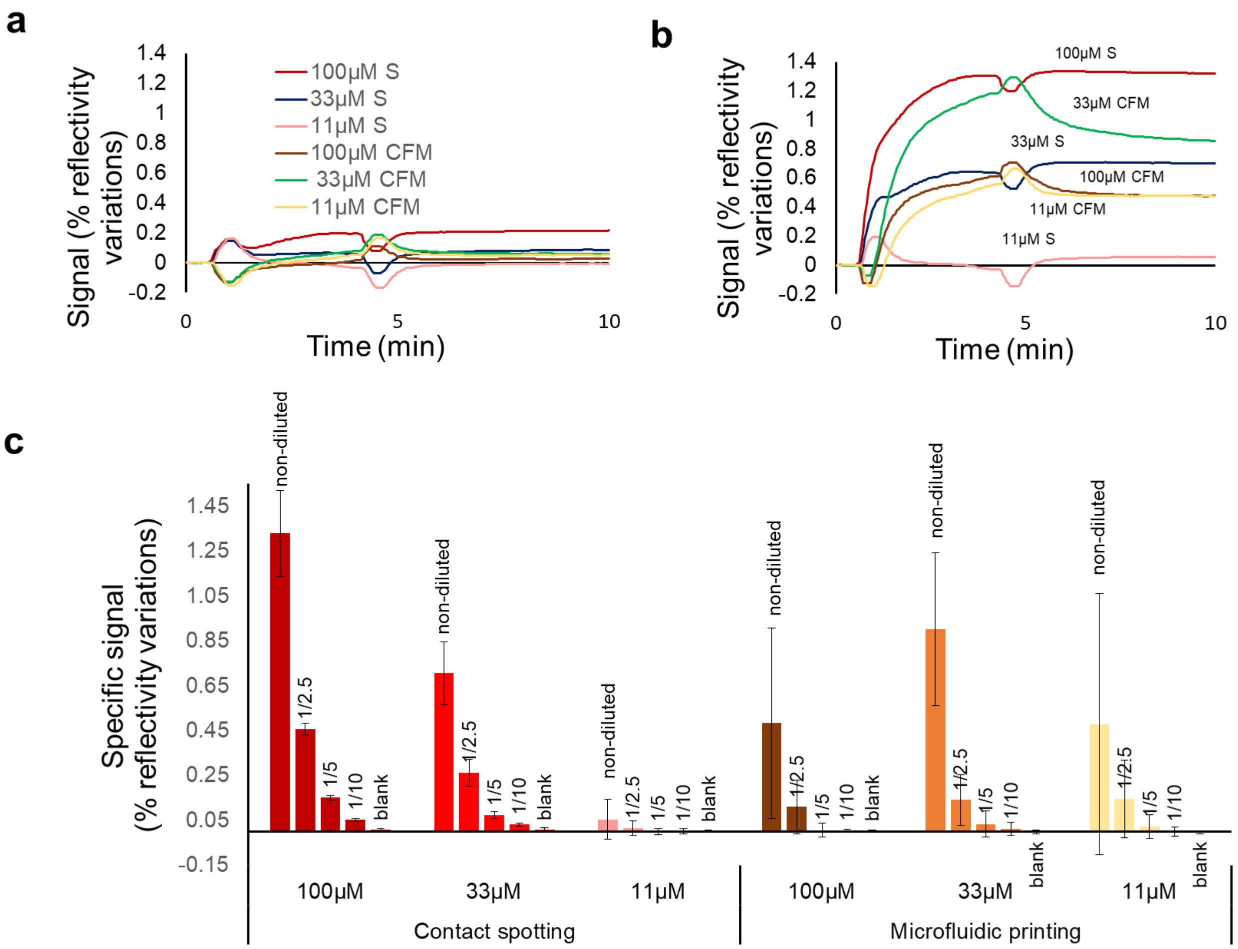

ESM Fig. 2 Optimization of surface plasmon resonance imaging method to measure glucose- human ADGRL1 binding. **(a)** Surface plasmon resonance imaging (SPRi) responses obtained from interaction between difference concentrations (μM, μmol/l) of biotinylated glucose conjugate and control cells (without ADGRL1) on two different immobilization systems: SPRi-Arrayer (micro-array format, S) and SPRi-CFM (continuous flow microspotter). **(b)** SPRi signal obtained from interaction between different concentrations (μM, concentration in μmol/l) of biotinylated glucose conjugate and ADGRL1 stably expressed in CHO cells on the two immobilization systems. **(c)** Dose dependent response obtained from interaction between different concentrations (μM, concentration in μmol/l) of biotinylated glucose and the ADGRL1 expressing CHO cells to select optimal conditions for downstream studying of glucose-ADGRL1 kinetics. These responses were normalized to the signals obtained with the biotinylated control conjugate without glucose. Non-diluted refers to 5 x10^5 cells/ml, which were then serially diluted 1/2.5, 1/5, 1/10 and 0 cells to produce a dose-dependent response. S, contact spotting by SPRi-Arrayer; CFM, continuous flow microspotter.

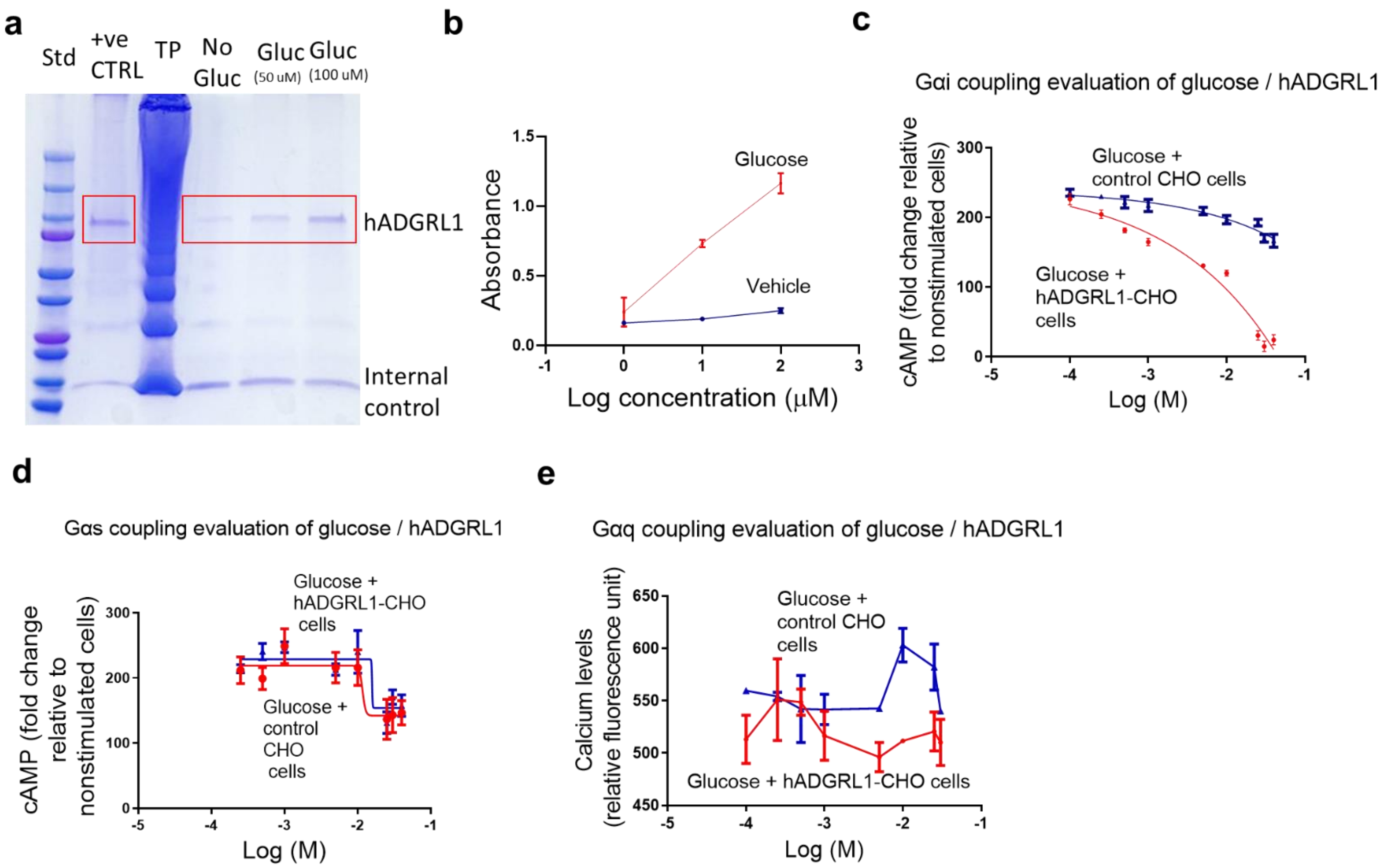

ESM Fig. 3 Regular (non-conjugated) glucose binds with recombinant human ADGRL1. (**a**) Gel electrophoresis and (**b**) Colorimetry. Glucose dose-dependently decreases cAMP in Gαi coupling assay (**c**) but not in Gαs coupling assay (**d**). Glucose doesn't change calcium levels in Gαs coupling assay (**e**), the assays were performed in duplicates. Std, standard protein ladder; +ve CTRL, recombinant human ADGRL1; TP, total protein left after purifying recombinant human ADGRL1; No Gluc, PBS (no glucose) + human ADGRL1 in Laemmli sample buffer; Gluc, glucose (regular non-conjugated) immobilized on epoxy-activated 96-plate wells + human ADGRL1 in Laemmli sample buffer; internal control, avidin; hADGRL1-CHO cells, stable CHO cells expressing human ADGRL1.

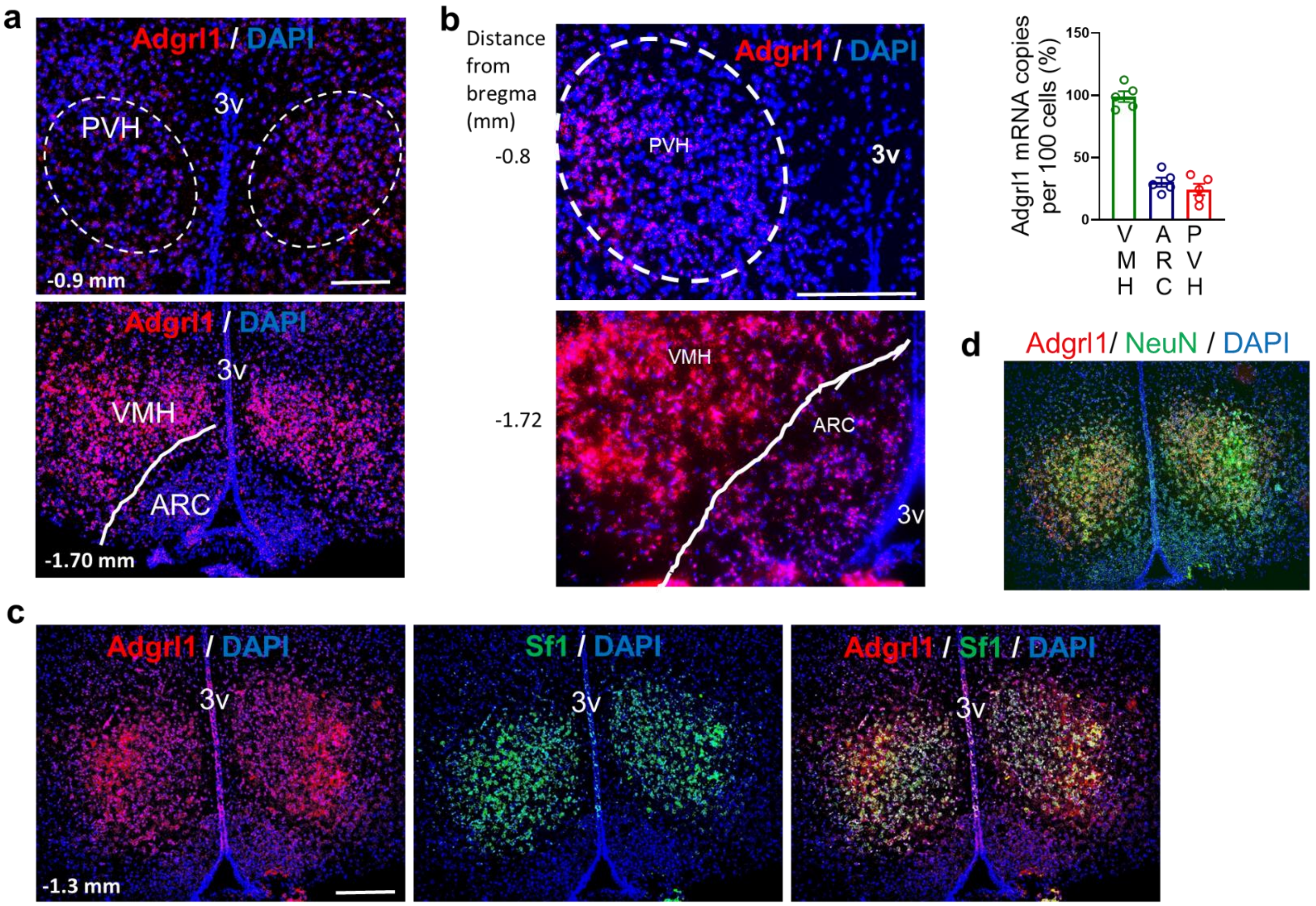

ESM Fig. 4 *Adgrl1* is highly expressed in the ventromedial nucleus of the hypothalamus. **(a)** Images from RNA in situ hybridization procedure demonstrating *Adgrl1* expression in the paraventricular (PVH), ventromedial (VMH), arcuate (ARC) nuclei of the hypothalamus, -0.9 and -1.70 mm are distances relative to the bregma. **(b)** 200x magnification of RNA in situ hybridization images of *Adgrl1* staining in the PVH, VMH, and ARC of the hypothalamus. Quantification of the staining is shown on the right, n= 4 sections per mouse were analyzed from 5 different mice. **(c, d)** Co-localization of *Adgrl1* with the VMH marker steroidogenic factor 1 (Sf1) and neuronal marker neuronal nuclei (NeuN), -1.3 mm is distance relative to the bregma. AP, anterior-posterior; ML, medial-lateral; DV, dorsal-ventral. Scale, 100 µm

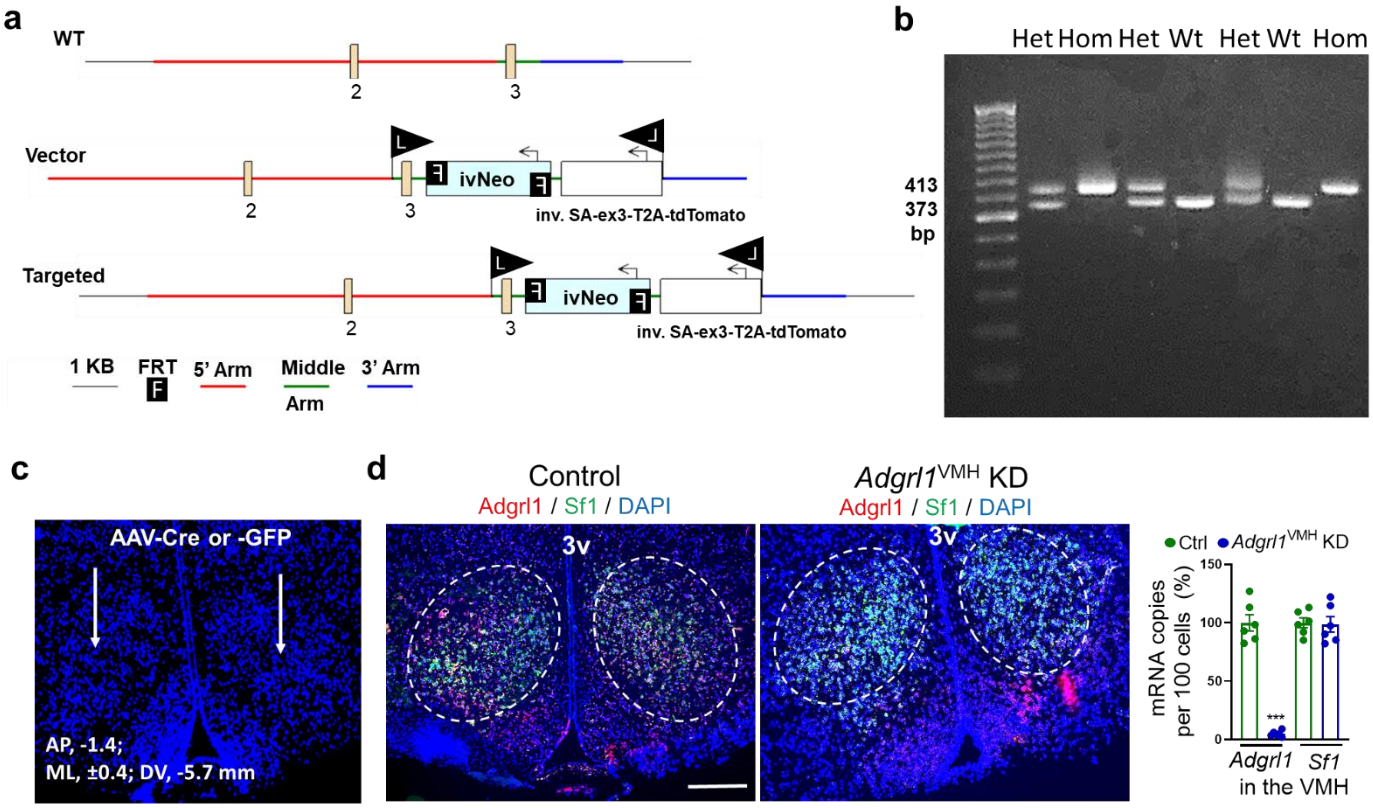

ESM Fig. 5 (a) Schematic diagram showing design of the vector that was used to produce floxed *Adgrl1* mice. **(b)** Representative genotypes of mice used in this study to produce *Adgrl1*$^{\mathrm{loxP/loxP}}$ homozygous (Hom, 413 bp band) mice. WT band is observed at 373 bp and bands at both the sizes indicated heterozygous (Het) mice. **(c)** Image shows the stereotaxic coordinates - relative to the bregma - we had used to inject adeno-associated viral (AAV) vector expressing Cre or GFP to induce deficiency of Adgrl1 specifically in the ventromedial nucleus of the hypothalamus (VMH). **(d)** Quantification of *Adgrl1* knockdown in the VMH (*Adgrl1*^{VMH} KD) after AAV-Cre injections, n=3 sections per mouse were analyzed at the end of the study (24 weeks after inducing the *Adgrl1* deficiency). The VMH area shown in white dotted circles was used for the quantification. Steroidogenic factor (Sf1) is a marker of the VMH and was used as an internal control. Two-tailed Unpaired Student's t-test, ***p<0.001.

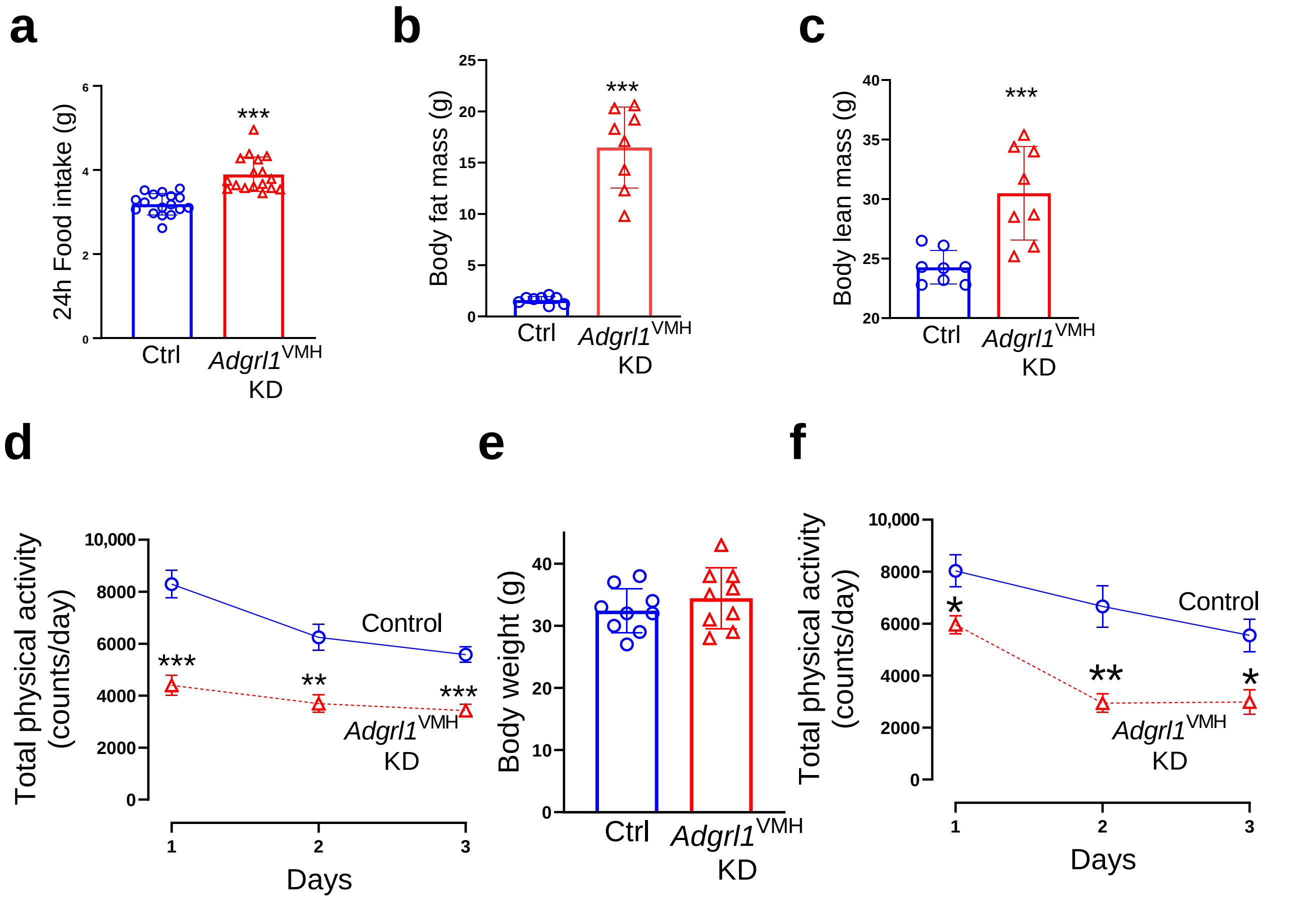

ESM Fig. 6 (a) Food intake, n=17 Ctrl and *Adgrl1^{VMH}* KD mice, **(b)** Body fat mass, **(c)** Body lean mass, **(d)** Total physical activity, n=8 Ctrl and *Adgrl1^{VMH}* KD mice, on the 21st week following *Adgrl1*^{VMH} knockdown (*Adgrl1*^{VMH} KD) in 29 weeks old male *Adgrl1^{VMH}* KD mice and their littermate controls. **(e)** Bodyweight, **(f)** Total physical activity in weight-matched mice on the 6th week following *Adgrl1*^{VMH} knockdown in 14 weeks old *Adgrl1^{VMH}* KD mice and their littermate controls, n=9 Ctrl and *Adgrl1^{VMH}* KD mice. Two-tailed Student's t-test or repeated measures two-way ANOVA followed by Bonferroni multiple comparison test were used for comparisons. *p<0.05, **p<0.01, and ***p<0.001. Data are shown as mean ± SEM. Ctrl, Control; *Adgrl1^{VMH}* KD*, Adgrl1* deficiency in the ventromedial nucleus of the hypothalamus.

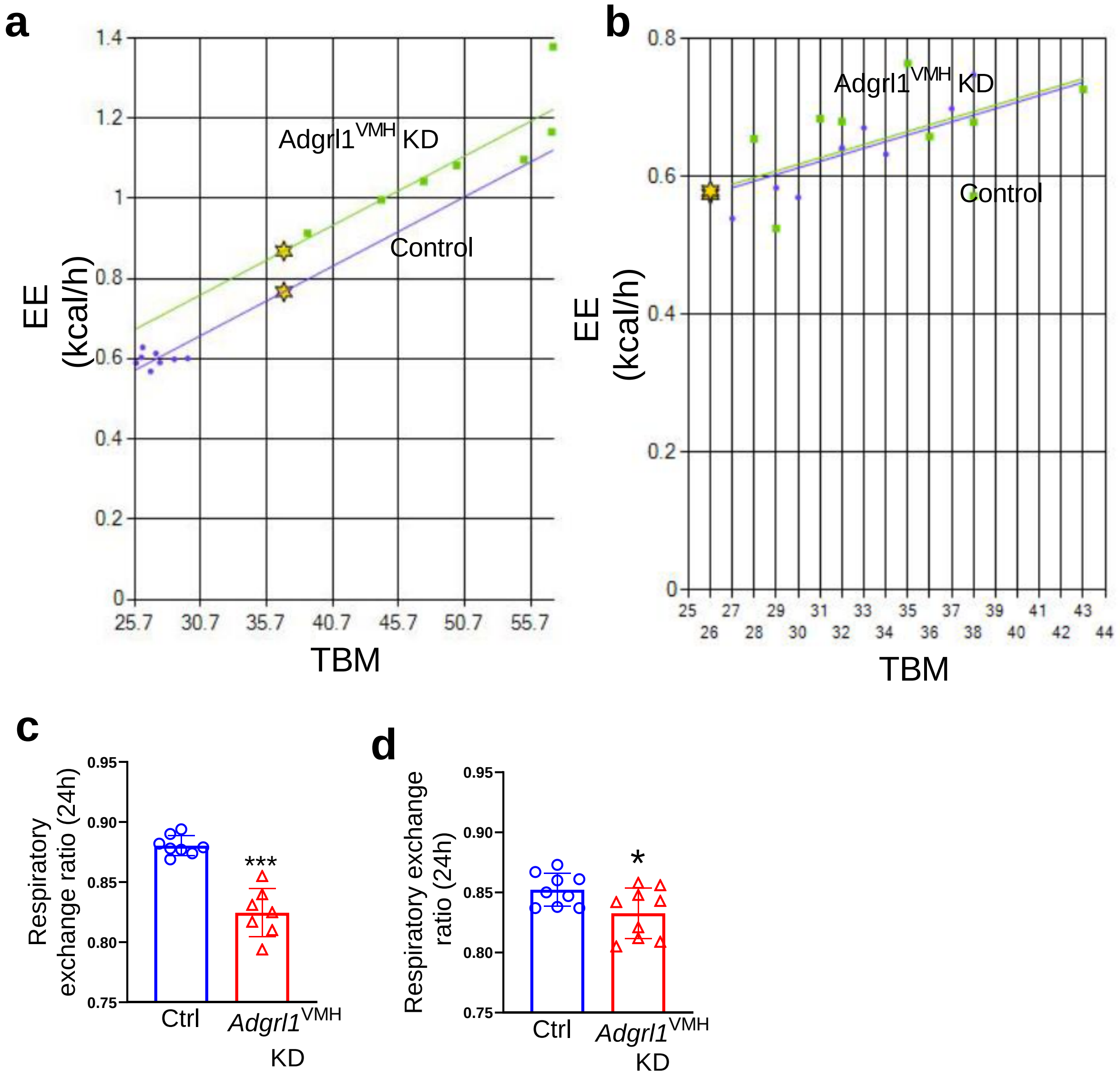

ESM Fig. 7 Normal energy expenditure in male mice with *Adgrl1* knockdown in the ventromedial nucleus of the hypothalamus (*Adgrl1^{VMH}* KD). (a, b) Energy expenditure (EE) analyzed using ANCOVA in obese **(a)** and weight-matched **(b)**, yellow star represents ANCOVA predicted EE at 37g **(a)** and 26g **(b)** total body mass (TBM). **(c, d)** Respiratory exchange ratio in obese **(c)** and weight-matched mice **(d).** Obese mice were analyzed on the 20th week following *Adgrl1*^{VMH} deficiency in 28 weeks old *Adgrl1^{VMH}* KD mice and their littermate controls. n=8 Ctrl and n=7 *Adgrl1^{VMH}* KD mice. Weight-matched mice were analyzed on the 6th week following *Adgrl1*^{VMH} knockdown in 14 weeks old *Adgrl1^{VMH}* KD mice and their littermate controls, n=9 Ctrl and *Adgrl1^{VMH}* KD mice. Two-tailed Student's t-test was used for comparisons. *p<0.05 and **p<0.001. Data are shown as mean ± SEM. Ctrl, Control; *Adgrl1^{VMH}* KD*, Adgrl1* knockdown in the ventromedial nucleus of the hypothalamus

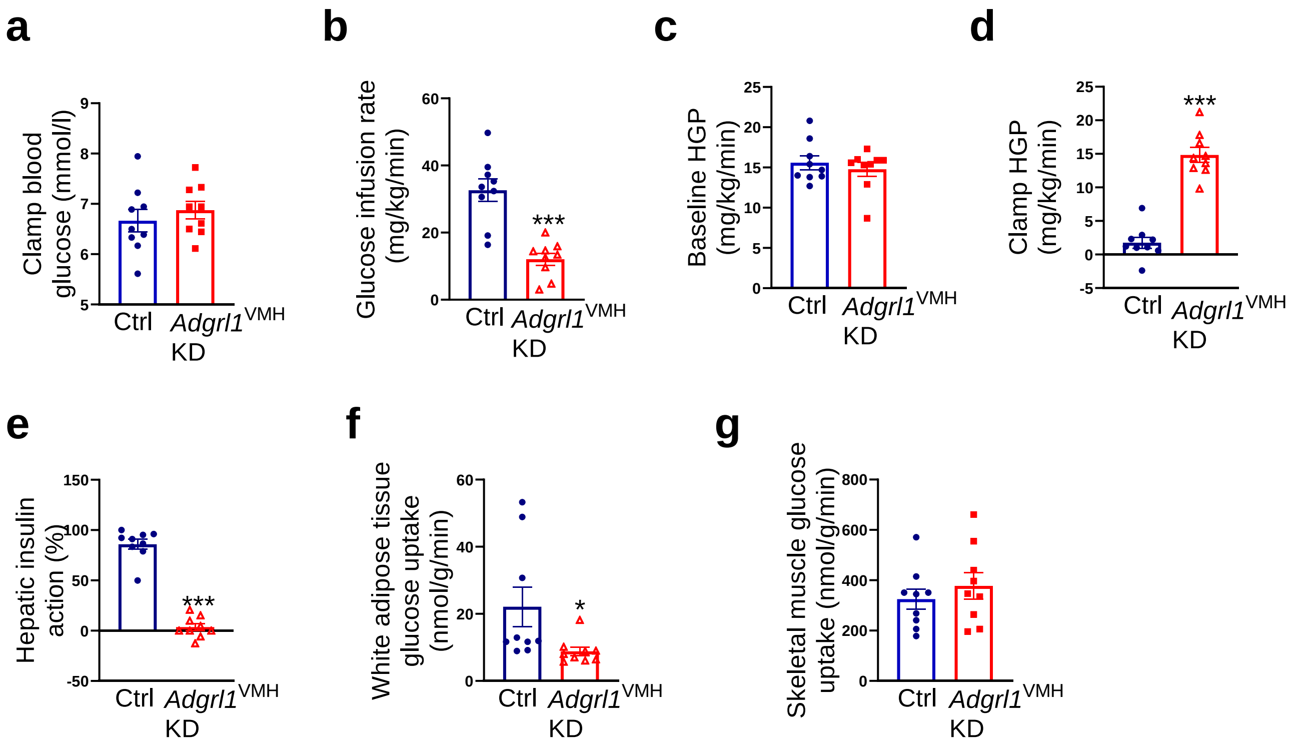

ESM Fig. 8 Hyperinsulinaemic-euglycaemic clamps in weight-matched male mice with *Adgrl1* knockdown in the ventromedial nucleus of the hypothalamus (*Adgrl1*^{VMH} KD). **(a)** Clamped blood glucose levels, **(b)** Glucose infusion rate (GIR), **(c,d)** Baseline **(c)** and clamp **(d)** hepatic glucose production (HGP), **(e)** Hepatic insulin sensitivity, **(f,g)** Glucose uptake in adipose tissue **(f)** and skeletal muscle **(g)** during hyperinsulinaemic-euglycaemic clamps on the 7th week following *Adgrl1*^{VMH} deficiency in 15 weeks old *Adgrl1*^{VMH} KD mice and their littermate controls, n=9 Ctrl and *Adgrl1*^{VMH} KD mice. Two-tailed Student's t-test *p<0.05 and ***p<0.001. Data are shown as mean ± SEM. Ctrl, Control littermates; *Adgrl1*^{VMH} KD, *Adgrl1* knockdown in the ventromedial nucleus of the hypothalamus.
